# Mitigation Strategies for Long-Term Corrosion in CFST Structures: A Systematic Review

**DOI:** 10.3390/ma19153330

**Published:** 2026-08-05

**Authors:** Safi Alsafi, Siti Aminah Osman, Faesal Alatshan, Abdullah Alghossoon, Azrul A. Mutalib

**Affiliations:** 1 Department of Civil Engineering, Faculty of Engineering and Built Environment, Universiti Kebangsaan Malaysia, Bangi 43600, Malaysia; azrulaam@ukm.edu.my; 2 Civil Engineering Department, College of Engineering Technology, Houn P.O. Box 61160, Libya; f.alatshan@ceh.edu.ly; 3 Civil Engineering Department, Faculty of Engineering, The Hashemite University, P.O. Box 330127, Zarqa 13133, Jordan; abdullahm_ab@hu.edu.jo

**Keywords:** CFST, external confinement, FRP, FRCM, hybrid systems, corrosion, structural performance, mitigation

## Abstract

Concrete-filled steel tube (CFST) structures are widely used in modern infrastructure due to their superior strength, ductility, and composite action. However, long-term corrosion of the steel tube, particularly under aggressive environmental conditions, poses significant challenges to their durability and structural performance. This study presents a comprehensive review of corrosion mechanisms and mitigation strategies for CFST structures. The primary corrosion processes, including general corrosion, localized (pitting) corrosion, and circumferential corrosion, are critically examined with emphasis on the influence of chloride ingress, carbonation, marine exposure, and combined environmental actions such as freeze–thaw cycles and sustained loading. The effects of corrosion on structural behavior are analyzed in terms of load-carrying capacity, ductility, buckling resistance, and failure modes. A systematic evaluation of existing mitigation strategies is conducted, encompassing material-based approaches, protective coatings, cathodic protection systems, and structural strengthening techniques such as fiber-reinforced polymer (FRP), fabric-reinforced cementitious matrix (FRCM), and steel jacketing. The comparative performance of these methods is assessed based on effectiveness, cost–benefit considerations, service life extension, and practical implement ability. The review highlights that no single mitigation strategy is universally optimal; instead, integrated approaches combining multiple techniques provide the most effective long-term protection. Key research gaps are identified in the areas of long-term performance monitoring, internal corrosion detection, and durability modeling under combined environmental actions. The findings of this study provide valuable insights for the design, maintenance, and rehabilitation of CFST structures, contributing to the development of more durable and sustainable infrastructure systems.

## 1. Introduction

Concrete-filled steel tubes (CFST) are composite structural members consisting of a hollow steel section infilled with concrete [1,2,3]. Through the synergistic interaction between the steel tube and the concrete core, CFST members exhibit superior mechanical and durability performance compared with conventional reinforced concrete and bare steel structures. The steel tube provides effective confinement to the concrete core, enhancing its compressive strength and ductility, while the concrete infill restrains local buckling of the steel tube and improves its load-bearing capacity. This composite action results in improved stiffness, energy dissipation capacity, and overall structural stability [4,5,6,7,8]. Due to these advantages, CFST systems have attracted significant attention in modern structural engineering and are increasingly considered a reliable solution for sustainable and resilient infrastructure development.

Another notable advantage of CFST structures is their enhanced fire resistance. During fire exposure, the concrete core acts as a thermal barrier, delaying heat transfer to the steel tube and reducing the rate of strength degradation [9,10]. In addition, the steel tube prevents spalling of the concrete core, thereby maintaining structural integrity under elevated temperatures. CFST members also demonstrate excellent seismic performance due to their high ductility, stable hysteretic behavior, and superior energy absorption capacity. These characteristics make CFST systems particularly suitable for structures located in earthquake-prone regions [11,12,13,14]. The combined benefits of strength, ductility, fire resistance, and seismic performance have contributed to the widespread implementation of CFST members in both building and bridge engineering applications.

Owing to these advantages, CFST structures have been widely adopted in various civil engineering applications, including high-rise buildings, long-span bridges, offshore platforms, port facilities, and transportation infrastructure [15,16,17,18]. In bridge engineering, CFST piers and arches are commonly employed to achieve high load capacity with reduced cross-sectional dimensions [17,18]. In marine and coastal environments, CFST piles and columns are increasingly used for wharves, jetties, and offshore foundations [15,16]. Furthermore, CFST systems are extensively utilized in seismic regions due to their excellent deformation capacity and damage tolerance. The growing application of CFST in critical infrastructure highlights the importance of ensuring their long-term structural safety and durability [19].

Despite their favorable mechanical properties, CFST structures remain susceptible to long-term deterioration, particularly due to corrosion of the steel tube. Corrosion in CFST members can occur on both external and internal surfaces, depending on environmental exposure and material characteristics. External corrosion is primarily caused by direct contact with aggressive environments, such as marine atmospheres, industrial pollutants, and de-icing salts. Internal corrosion, although less visible, may occur due to moisture penetration, chemical reactions within the concrete core, and the presence of dissolved oxygen and chlorides [2,20,21]. Among the various deterioration mechanisms affecting CFST structures, corrosion is considered one of the most critical factors governing long-term durability and service life performance.

Chloride ingress represents one of the most critical factors contributing to corrosion in CFST structures. Chloride ions can penetrate through micro cracks, pores, and defects in the concrete infill and accumulate at the steel–concrete interface, leading to depassivation of the protective oxide film on the steel surface. Once this passive layer is destroyed, localized corrosion processes, such as pitting corrosion, may initiate and propagate rapidly. In coastal and offshore environments, airborne salt spray and seawater exposure significantly accelerate chloride-induced corrosion [22,23]. The severity of chloride-induced corrosion becomes particularly critical in marine and coastal infrastructures where CFST members are continuously exposed to aggressive saline environments.

Carbonation of concrete is another major mechanism affecting the durability of CFST structures [24]. Carbon dioxide from the atmosphere diffuses into the concrete core and reacts with alkaline hydration products, reducing the pH of the pore solution [24]. This pH reduction weakens the protective environment surrounding the steel tube, increasing its vulnerability to corrosion. In addition, repeated wetting-drying cycles, commonly encountered in tidal zones and fluctuating groundwater conditions, promote the transport of aggressive ions and oxygen, thereby accelerating corrosion reactions [24,25]. The combined effects of chloride ingress, carbonation, and environmental exposure significantly accelerate the deterioration process in CFST members under long-term service conditions.

Marine exposure further intensifies the deterioration of CFST members through combined chemical, physical, and biological actions [26]. Salinity, temperature variations, biofouling, and mechanical abrasion jointly contribute to material degradation. The interaction between corrosion products and the confined concrete core may induce internal stresses, leading to cracking, debonding, and loss of composite action. Over time, these degradation mechanisms compromise the load-bearing capacity, ductility, and serviceability of CFST structures [26,27,28].

The progressive corrosion of CFST structures poses significant concerns regarding service life, structural safety, and economic sustainability [29,30]. As many CFST bridges, marine facilities, and high-rise buildings are designed for service lives exceeding 50 to 100 years, premature corrosion-related damage may result in unexpected performance deterioration and costly rehabilitation. In severe cases, corrosion-induced section loss and bond degradation can trigger brittle failure modes, threatening public safety [31,32]. Consequently, ensuring the durability and corrosion resistance of CFST structures has become an important consideration in modern infrastructure design and asset management.

Increasing demands for durability and resilience in modern infrastructure further emphasize the necessity of effective corrosion mitigation strategies [33]. With growing urbanization, climate change impacts, and environmental pollution, CFST structures are increasingly exposed to aggressive service conditions [34,35]. Therefore, contemporary design approaches increasingly emphasize life-cycle performance, durability-based design principles, and proactive corrosion prevention measures to ensure long-term structural reliability. Instead, durability-oriented design and proactive corrosion control measures are essential for achieving sustainable infrastructure development [36,37].

Maintenance and retrofit of corroded CFST structures present considerable technical and economic challenges [38]. The enclosed nature of the steel tube and concrete core makes inspection and repair of internal corrosion particularly difficult. Conventional maintenance techniques, such as surface coating renewal or cathodic protection installation, may be costly and disruptive, especially for in-service bridges and offshore facilities. Moreover, retrofitting measures must be compatible with existing structural systems and operational constraints. These challenges necessitate the development and systematic evaluation of reliable, cost-effective, and practical corrosion mitigation solutions [39,40].

Several review studies have previously investigated the structural behavior, design performance, and application of CFST structures. Han et al. [41] presented a comprehensive review on the development and advanced applications of CFST members, emphasizing their mechanical performance and structural efficiency. Subsequent review studies focused on specialized aspects of CFST systems, including stiffened CFST members [42], CFST truss girders [43], high-strength CFST behavior [44], and hybrid strengthening techniques involving steel fibers and glass fiber-reinforced polymer (GFRP) jacketing [45]. In addition, extensive reviews have been conducted on the fire performance [46], seismic behavior [47], and sustainability-related applications of CFST systems incorporating recycled materials [48,49]. These studies significantly contributed to the understanding of structural behavior, material performance, and design advancements of CFST structures. However, despite the increasing concern regarding durability degradation in aggressive environments, existing review studies have primarily concentrated on structural behavior and mechanical performance, while limited attention has been given to long-term corrosion mechanisms and mitigation strategies in CFST structures. In particular, a comprehensive systematic review addressing chloride-induced corrosion, carbonation effects, marine exposure, corrosion prevention techniques, durability-oriented design approaches, and maintenance strategies for CFST systems is still lacking in the current literature. Therefore, there remains a clear need for a dedicated systematic review on mitigation strategies for long-term corrosion in CFST structures to support the development of durable, resilient, and sustainable composite infrastructure systems.

In response to the increasing durability concerns associated with CFST structures, this systematic review provides a comprehensive synthesis of existing studies on long-term corrosion mechanisms and mitigation strategies for CFST members. The review critically examines the dominant deterioration mechanisms under various environmental conditions, including chloride exposure, carbonation, marine environments, and cyclic wetting-drying actions. Furthermore, available mitigation approaches are systematically evaluated, including corrosion-resistant materials, high-performance concrete, corrosion inhibitors, surface protection systems, electrochemical techniques, and durability-oriented structural design strategies. Finally, current research gaps, limitations in experimental and numerical investigations, and challenges associated with field implementation are identified to provide recommendations for future research and practical applications aimed at enhancing the long-term durability and sustainability of CFST infrastructure systems.

## 2. Methodology

This systematic review was conducted following the Preferred Reporting Items for Systematic Reviews and Meta-Analyses (PRISMA 2020 [50]) guidelines to ensure a transparent, reproducible, and comprehensive literature review process. The completed PRISMA 2020 Checklist is provided in the Supplementary Materials. The review aimed to identify, evaluate, and synthesize published studies addressing the long-term corrosion mechanisms affecting concrete-filled steel tube (CFST) structures and the corresponding mitigation strategies developed to enhance their durability and structural performance.

This systematic review was not prospectively registered in PROSPERO or any other systematic review registry.

A comprehensive literature search was conducted using six major scientific databases, including ScienceDirect, Scopus, Web of Science, SpringerLink, Taylor & Francis Online, and the ASCE Library. These databases were selected because they provide extensive coverage of structural engineering, construction materials, corrosion science, and infrastructure durability research. The search employed combinations of keywords related to CFST structures, corrosion mechanisms, durability, deterioration, rehabilitation, and mitigation strategies using Boolean operators (AND/OR). The adopted search strategy is summarized in Table 1. Only peer-reviewed publications written in English were considered.

The literature search focused on publications up to January 2025. The literature search was conducted using Boolean operator searches (AND/OR) and the keywords indicated in Table 1. One example of a search query was: (“Concrete-filled steel tube” OR CFST OR “Concrete-filled steel tubular column”) AND (corrosion OR durability OR deterioration OR chloride OR carbonation OR pitting) AND (mitigation OR protection OR rehabilitation OR strengthening OR repair). The search syntax was modified to suit individual databases where necessary without altering the search logic.

The retrieved publications were screened according to predefined eligibility criteria. Studies were included if they investigated corrosion mechanisms, durability, deterioration processes, or mitigation strategies related to CFST members through experimental, numerical, or analytical approaches. Peer-reviewed journal articles and conference proceedings published in English were considered eligible. Editorials, book chapters, technical notes, duplicate publications, and studies focusing solely on structural behavior without addressing corrosion-related aspects were excluded. Although the systematic review primarily focused on conventional carbon-steel CFST members, supporting studies involving externally bonded strengthening systems, such as fiber-reinforced polymer (FRP), fabric-reinforced cementitious matrix (FRCM), and steel jacketing, were incorporated into the narrative discussion when they provided relevant evidence regarding corrosion mitigation or rehabilitation of CFST structures.

**Figure 1:**
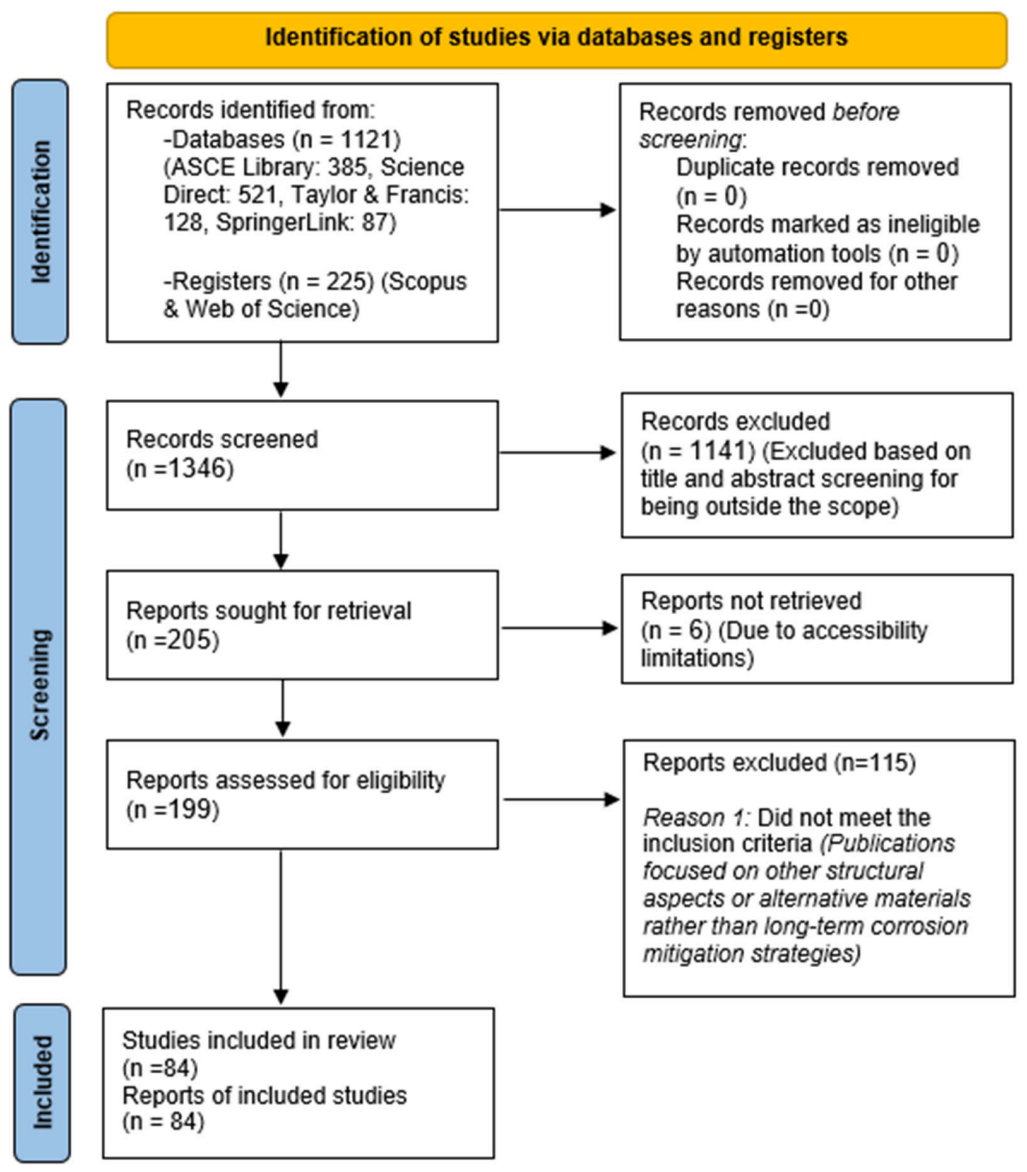
The adopted systematic review PRISMA flow diagram.

The study selection process followed the PRISMA 2020 framework and comprised four sequential stages: identification, screening, eligibility assessment, and final inclusion. Initially, duplicate records were removed, followed by title and abstract screening to eliminate publications outside the scope of this review. The remaining studies underwent full-text assessment according to the predefined eligibility criteria before final inclusion. The complete screening and selection procedure is illustrated in the PRISMA flow diagram presented in Figure 1.

For each eligible publication, relevant information was systematically extracted, including the type of CFST member, exposure environment, corrosion mechanism, corrosion simulation method, mitigation strategy, research methodology, principal findings, and reported limitations. The extracted information was subsequently synthesized to compare the effectiveness of different corrosion mitigation approaches and to identify current research trends, knowledge gaps, and future research needs in the field of CFST durability. The extracted characteristics of all included studies are presented in Table 2.

This review is subject to several limitations. Only English language publications indexed in the selected databases were included. Furthermore, considerable variability exists among the reviewed studies regarding specimen configurations, environmental exposure conditions, accelerated corrosion techniques, testing procedures, and evaluation methods, limiting direct quantitative comparison. In addition, relatively few long-term field investigations have been reported for corrosion mitigation in CFST structures, highlighting the need for further experimental and in-service validation of existing mitigation strategies.

## 3. Characteristics of the Included Studies

A total of 84 studies satisfied the predefined eligibility criteria and were included in the qualitative synthesis. The extracted information comprised the intervention type, comparison or control (where applicable), outcomes measured, and the principal conclusions of each study. The characteristics of the included studies are summarized in Table 2, providing an overview of the research approaches and mitigation strategies investigated for improving the long-term durability of concrete-filled steel tube (CFST) structure.

## 4. Overview of Concrete-Filled Steel Tube (CFST) Structures

Concrete-filled steel tube (CFST) members combine the complementary mechanical properties of structural steel and concrete, resulting in a highly efficient composite structural system. The steel tube provides confinement to the concrete core, enhancing its compressive strength and ductility, while the concrete infill delays local buckling of the steel tube and improves its compressive stability [41,51,52]. Owing to this composite interaction, CFST members exhibit higher load-bearing capacity, improved deformation capacity, and superior energy absorption compared with conventional reinforced concrete and hollow steel members.

CFST columns are commonly manufactured using circular (CHS), square (SHS), and rectangular (RHS) hollow steel sections, although other cross-sectional configurations, including polygonal and elliptical sections, have also been developed for architectural and structural applications [41]. Among these geometries, circular sections generally provide the highest confinement efficiency, whereas square and rectangular sections are more susceptible to local buckling because of their flat plate elements.

**Figure 2:**
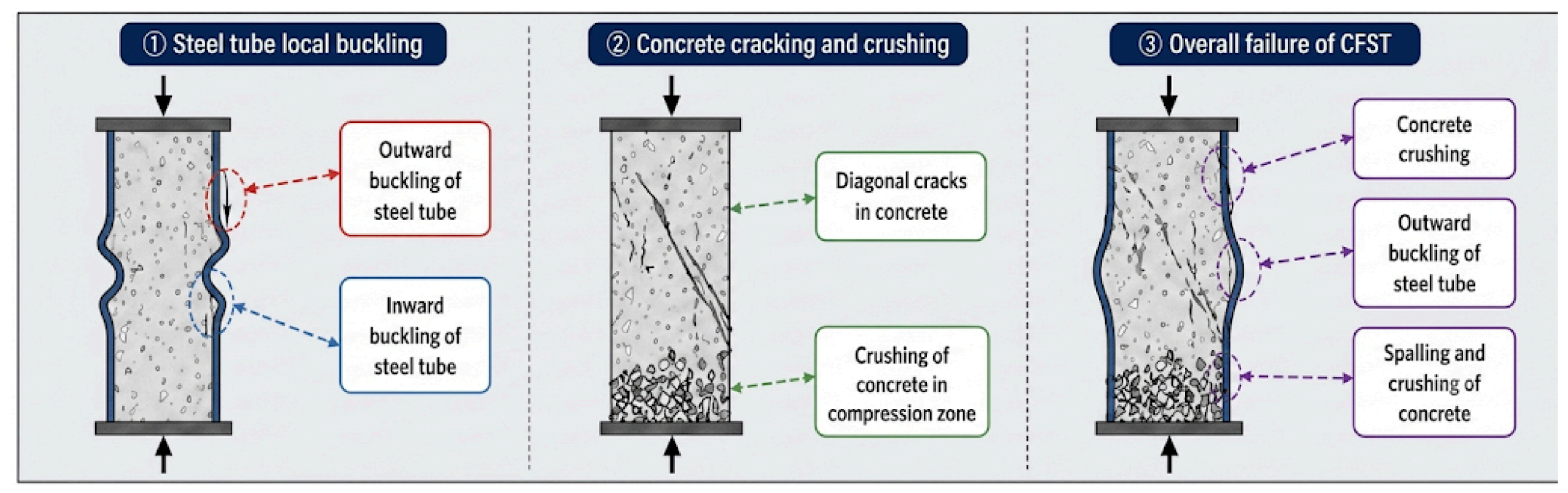
Schematic failure modes of hollow steel tube, concrete and CFST stub columns. Illustrated by the authors based on Ref. [41].

Experimental studies have demonstrated that the composite interaction between the steel tube and concrete core significantly improves structural performance. Unlike hollow steel tubes, which may buckle inward and outward, or plain concrete columns, which typically fail in a brittle manner, CFST members mainly exhibit outward local buckling accompanied by a more ductile failure of the confined concrete core (Figure 2). Consequently, CFST members achieve greater strength, ductility, and structural resilience than either constituent material acting independently [41].

Despite these structural advantages, the exposed steel tube remains vulnerable to corrosion when subjected to aggressive service environments, including marine, industrial, and chloride-rich conditions. Corrosion progressively reduces the steel wall thickness, weakens the confinement effect, and ultimately degrades the composite behavior of CFST members. Therefore, improving the long-term durability of CFST structures through effective corrosion mitigation strategies has become a major research focus. Furthermore, externally bonded strengthening systems, such as fiber-reinforced polymer (FRP) composites, have been increasingly investigated to enhance both corrosion resistance and structural performance of deteriorated CFST members [28,39,40].

## 5. Long-Term Corrosion Mechanisms in CFST Structures

Corrosion in CFST structures develops through various mechanisms influenced by environmental exposure, chloride ingress, moisture, and mechanical actions. These deterioration processes range from uniform and localized corrosion to more complex forms associated with marine environments, sustained loading, freeze–thaw cycles, and acid rain. The following subsections review the principal corrosion mechanisms and their effects on the long-term structural performance of CFST members.

As opposed to ordinary steel constructions, CFST member corrosion occurs from a combination of different processes which take place at the steel surface, steel–concrete interface, and enclosed concrete core. The corrosion process involves not only environmental conditions but oxygen availability, moisture pathways, presence of chlorides, and steel–concrete interface conditions. Whereas external corrosion starts at the steel surface exposed to the marine atmosphere, industry pollution, and de-icing salts, internal corrosion takes place at the localized regions where moisture and aggressor ions penetrate inside the enclosed system through tube ends, poor workmanship, welds, cracks, and other openings. Whereas the concrete core provides an alkaline medium, which causes steel passivation initially, local depassivation can start if chloride ions are accumulated at the steel–concrete interface region or due to carbonation, leading to the pH of the pore solution decreasing. Unlike ordinary reinforced concrete, carbonation in CFST members is mainly conducted through the ingress pathways and not through the uniform ingress into the exposed concrete cover due to the enclosure of the concrete core inside the surrounding steel tube. In addition, the oxygen availability within CFST members is typically lower than that of reinforced concrete, which can prevent the onset of corrosion; nevertheless, once there is penetration of oxygen and chlorides through the defects, corrosion can still proceed despite its difficulty in being detected during regular inspections. As such, the process of corrosion within CFST members is controlled by the interaction of the environment, materials, defects, and transportation processes, which results in the heterogeneous nature of its corrosion process.

### 5.1. General Corrosion

General corrosion is the most common form of deterioration in CFST structures, characterized by relatively uniform material loss over large areas of the steel surface. Although often less severe than localized corrosion in terms of stress concentration, its progressive nature can significantly reduce the steel tube thickness and compromise the long-term structural performance of CFST members [21,53]. The following subsections discuss the effects of uniform wall thinning and marine atmospheric exposure on corrosion development.

#### 5.1.1. Uniform Wall Thinning

General (uniform) corrosion in CFST members results in relatively even steel wall thinning across the exposed surface due to continuous electrochemical oxidation processes at the steel–electrolyte interface [21,54]. In this process, the tube wall thickness is gradually lowered, which causes the effective cross-sectional area to be reduced and consequently decreases the axial compressive strength of CFST elements. Even though uniform corrosion does not create as serious stress concentration as localized corrosion does, its effects become more important as the exposure period increases, especially for thin-walled CFST elements. In such situations, even small changes in tube wall thickness can considerably increase the (D/t) ratio, decrease stability, and lessen the confinement efficiency provided by the tube surrounding the concrete core [21,54,55]. The experimental studies have indicated that gradual uniform corrosion changes the failure mode of the element from ductile shear bulge failure to instability-dominated failure modes [56]. Therefore, the structural implications of uniform corrosion depend not only on the mass of material lost but also on the period of exposure and geometric properties of the steel tube, stressing the significance of taking into account the process of corrosion when assessing the performance of CFST members.

#### 5.1.2. Marine Atmospheric Effects

Marine environments are highly aggressive for CFST structures due to the combined effects of moisture, chloride ions, oxygen, and temperature fluctuations, leading to significantly higher corrosion rates than in inland conditions [15,56]. In marine atmospheric zones, salt spray deposits chloride ions on the steel surface, where they accumulate in surface defects and break down protective oxide layers, initiating corrosion processes [57]. Moisture sustains these electrochemical reactions, while temperature fluctuations enhance both corrosion activity and surface condensation. Field investigations have reported severe atmospheric deterioration in coastal CFST members after approximately 10–15 years of exposure, with corrosion rates typically ranging from 10 to 25 mm/year [58], depending on the local environmental conditions and exposure severity. In these environments, corrosion predominantly initiates on the external steel surface, whereas the concrete core continues to provide an alkaline environment that delays internal deterioration until aggressive agents penetrate through localized defects or discontinuities [55], These observations prove that the level of general corrosion on CFST structures highly depends on the exposure environment in which they are located, and therefore, any corrosion rate and level of corrosion damage that have been recorded should always be understood within their respective environments.

### 5.2. Localized and Circumferential Chloride Corrosion

Localized and circumferential chlorides attack is among the most important deterioration forms occurring in CFST elements due to their significant spatial non-uniformity resulting in increased stress concentration and accelerated structural damage. Unlike uniform corrosion which leads to relatively even wall thinning, localized corrosion causes disruption of the integrity of the steel tube and hence facilitates early local buckling, cracking, and loss of load-bearing capacity. This type of corrosion is especially dangerous in the case of exposure of the structure to the aggressive marine environment, where the processes of chloride penetration and passivity film destruction are intensified due to cyclic wetting-drying processes. Thus, in addition to the amount of corrosion, its location, circumferential nature, and depth play an important role. The next subsections address the effects of splash zone exposure and circumferential patterns of corrosion on the behavior of CFST elements.

#### 5.2.1. Splash Zone Effects

The splash zone, typically located at a height of 1 to 10 m above the water surface, is among the harshest zones of exposure for CFST structures, as it is continuously subjected to cyclic wet–dry cycles and high chloride deposition [58]. During the wetting phase, the seawater acts as the source of chloride ions for the steel surface, allowing electrochemical reactions of corrosion to proceed. In the next phase of drying, evaporation leads to higher concentrations of chlorides around the steel surface, thus making it more corrosive [59]. Wetting and drying cycles further worsen this process through the formation of corrosion byproducts, swelling, and differential shrinking, all contributing to steel deterioration, while making corrosion more likely to occur in CFST sections.

Xie et al. [60] examined CFST beams under exposure to simulated acid rain and splash zones, where higher rates of corrosion, two to three times that of atmospheric and underwater environments, were observed. The synergistic influence of wave action, wet and dry cycling, temperature cycling, and chemical attack resulted in extremely aggressive exposure to the environment leading to pitting initiation and general corrosion.

#### 5.2.2. Mid-Height Circumferential Attack

**Figure 3:**
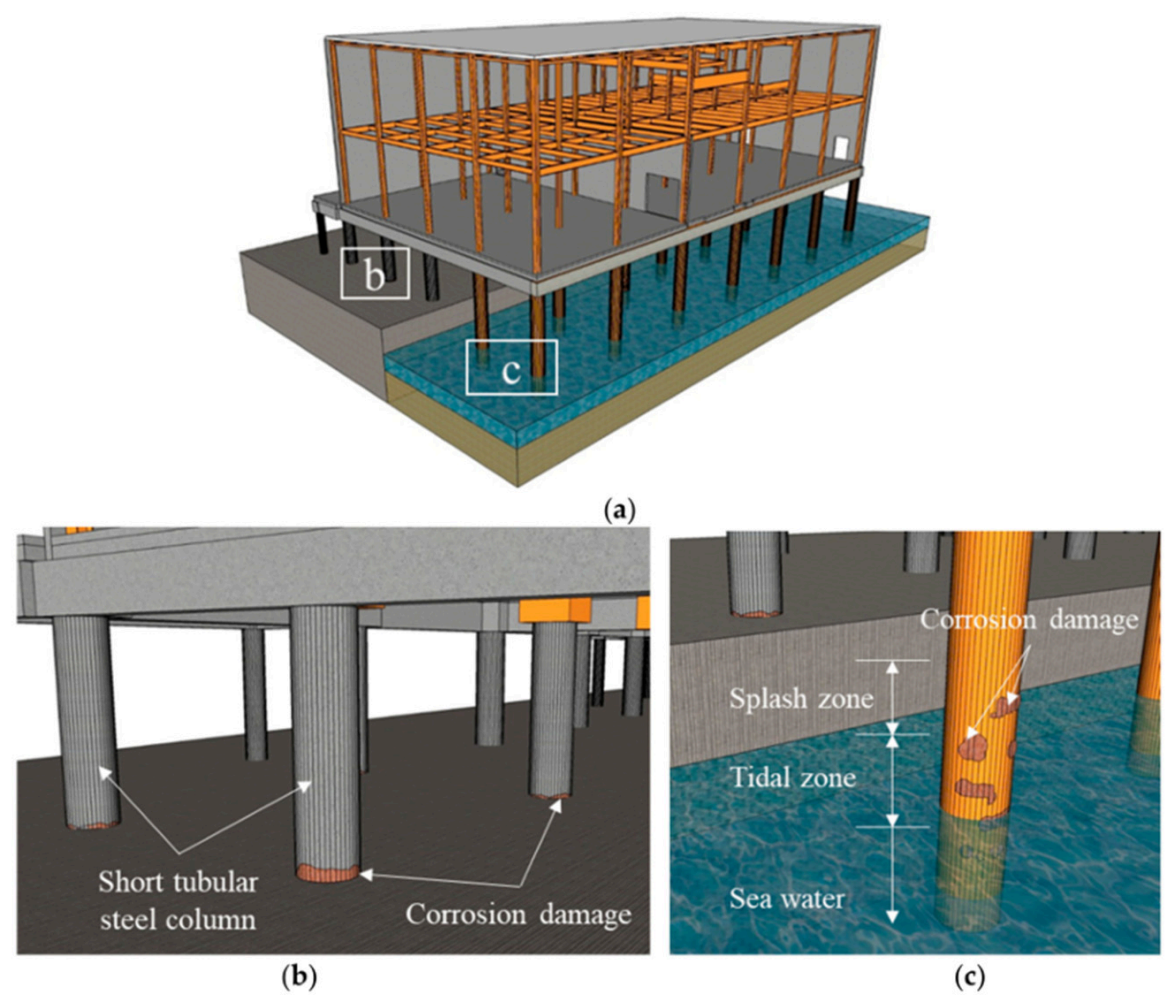

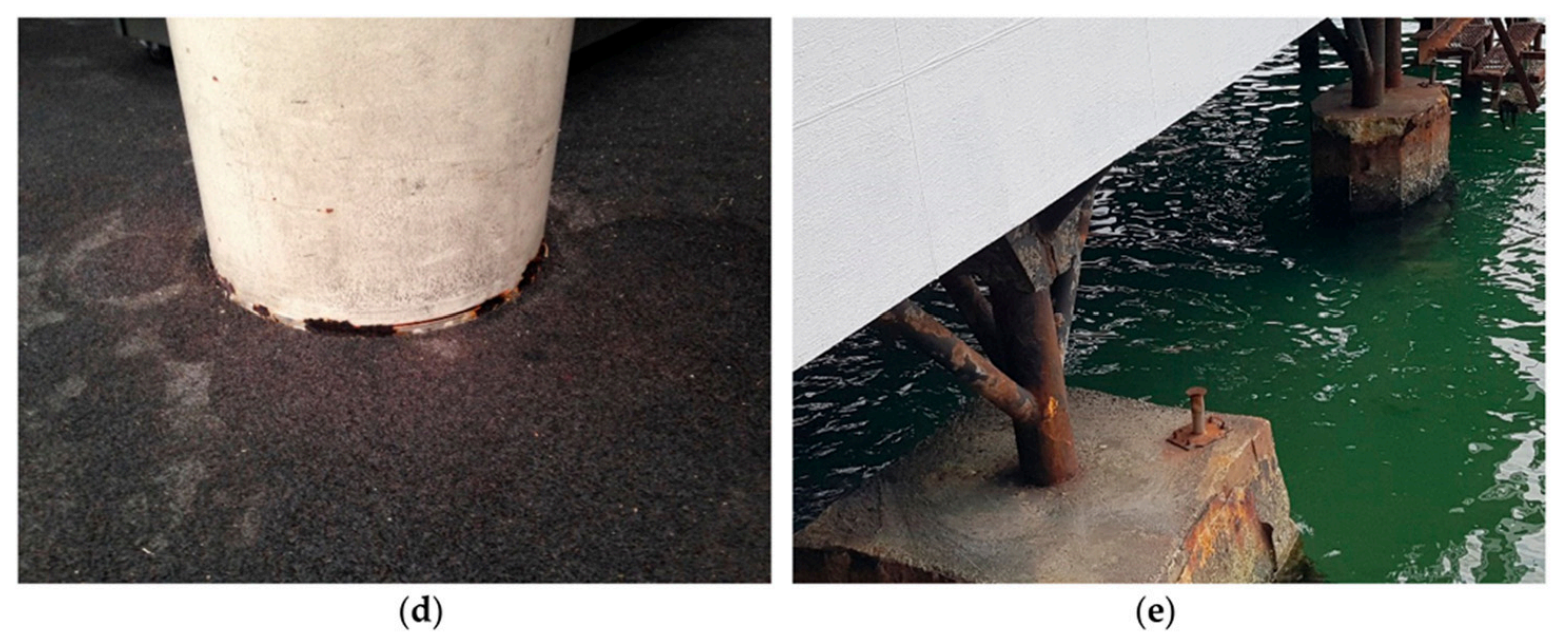
Schematic and field observations of corrosion in short tubular steel columns: (**a**) different offshore exposure environments; (**b**) localized corrosion at the ends; (**c**) corrosion along the column length; (**d**) observed end corrosion; and (**e**) observed longitudinal corrosion [57].

Circumferential corrosion patterns in CFST structures often develop preferentially at mid-height locations, particularly in vertical members subjected to variable environmental exposure [21,61]. The selective corrosion of the material has a relationship with moisture retention and chloride build-up around the steel pipe, whereby the environment here tends to be more aggressive compared to that of the areas that are continuously submerged, but different from that of areas that are totally exposed to the atmosphere. Such an environment leads to uneven corrosion of the pipe, making it vulnerable to local buckling [62]. Corrosion height significantly influences the compressive strength of steel tubular columns and should be evaluated together with corrosion depth and circumferential extent [57]. Since corrosion damage commonly develops near the mid-height of columns, the effects of damage location under different exposure conditions should also be considered (Figure 3a). In contrast, local corrosion often occurs at column ends due to the accumulation of moisture and debris, which accelerates deterioration (Figure 3b,d). Furthermore, environmental exposure and coating degradation play important roles in corrosion propagation and should be accounted for in durability assessments (Figure 3c,e).

Experimental studies on artificially fabricated localized corrosion damage in short tubular columns revealed that residual compressive strength decreased substantially with corrosion depth, height, and circumferential extent [57]. When the amount of corrosion exceeded about half the member buckling wavelength, the load-carrying capacity was quickly lost due to local buckling tendencies. In addition, the circumferential extent of corrosion had a large impact on structure behavior. Full circumferential corrosion formed a constant weak zone which would act as a stress concentration point, while partial circumferential corrosion led to a less damaging effect on the structure, depending on its orientation with respect to the principle stress direction.

#### 5.2.3. Pitting and Notch-Type Defects

Pitting corrosion is one of the most critical localized degradation mechanisms in CFST structures exposed to chloride-rich environments. It initiates when chloride ions locally disrupt the protective passive film on the steel surface, leading to highly localized anodic dissolution [21,41,63,64,65]. Once initiated, pits propagate autocatalytically as hydrolysis reactions create acidic conditions within the cavity, accelerating localized metal dissolution. In CFST members, particularly in marine splash zones, long-term field exposure studies conducted under severe marine conditions have reported pit depths of approximately 4–6 mm after 10–15 years of service. These localized defects significantly reduce structural performance by acting as severe stress concentrators, with stress concentration factors typically ranging from 2.5 to 4.0 depending on pit geometry [20]. In CFST systems subjected to axial and cyclic loading, pitting accelerates fatigue damage and premature yielding by reducing the effective steel area, increasing local stress demand, and promoting crack initiation. Although overall section loss may appear moderate, the structural impact is highly non-linear; experimental and numerical studies have demonstrated that there could be losses due to corrosion ranging from 15% to 20% which can lead to up to 40% to 50% loss in axial load-carrying capacity due to combined effects of localized pitting and defect clustering [63].

### 5.3. Corrosion Under Combined Actions

In practice, corrosion in CFST structures rarely occurs in isolation and is often accompanied by mechanical and environmental actions that accelerate deterioration. Interaction of the aforementioned factors can cause changes in the corrosion kinetics, increase the rate of material degradation, and deteriorate the performance of CFST members more than that resulting from any single factor acting alone. The following subsections present the main coupled mechanisms of deterioration described in the literature and the impact of such mechanisms on the durability and load-carrying capacity of CFST structures.

#### 5.3.1. Corrosion + Sustained Loading

However, the relationship between corrosion and mechanical loads is a crucial durability issues in CFST constructions due to the effect that mechanical stresses have on corrosion and, at the same time, corrosion impacts the structure’s stability through reducing the capacity of the steel tube. According to some researchers, sustained tensile and compressive stresses increase anodic dissolution and cause stress-induced corrosion in the presence of chloride ions [64,65]. Experimental studies on circular CFST specimens subjected to coupled sustained tensile loading and accelerated chloride corrosion have shown complex time-dependent degradation behavior, characterized by progressive reductions in strength and stiffness. Members that were concurrently loaded had higher degrees of damage when compared with those only exposed to corrosion during the same period, thus pointing out the synergy between corrosion and loading conditions [65].

#### 5.3.2. Corrosion + Freeze–Thaw Cycling

Freeze–thaw cycling combined with corrosion forms a severe coupled deterioration mechanism, particularly in cold climates and high-altitude regions where CFST structures are frequently exposed to repeated thermal fluctuations and moisture ingress [27]. When water penetrates micro cracks, pores, or corrosion-induced pits and subsequently freezes, the resulting ~9% volumetric expansion of ice generates internal tensile stresses that may exceed the tensile capacity of surrounding concrete, leading to cracking, surface spalling, and accelerated degradation of the concrete core [66,67].

He et al. [68] examined square CFST stub columns after freeze–thaw exposure and observed that bearing capacity decreases by over 30%, with damage increasing as cycle number rises, while ductility changes remain within 10%. The results also indicate that both AISC 360–16 and EC4 provide conservative predictions of ultimate capacity. Lyu et al. [69] investigated CFST circular stub columns under combined corrosion and freeze–thaw cycles. Results show shear failure with significant degradation of residual capacity and ductility as corrosion and cycle effects increase, supported by a validated FE model and a simplified capacity prediction method.

#### 5.3.3. Corrosion + Acid-Rain Exposure

Acid rain represents a significant environmental threat to CFST structures in industrial regions and areas downwind of pollution sources. Acid rain, typically with pH values between 4 and 5, accelerates corrosion rates by disrupting passive oxide layers and creating acidic microenvironments at the steel surface [60].

Experimental investigations on CFST members subjected to simulated acid rain exposure revealed yield strength reductions of 15–30% and ultimate tensile strength reductions of 10–25% depending on acid rain severity and exposure duration. When combined with mechanical loading, ultimate moment capacity of CFST beams decreased by 15–40% over 20-year simulated exposure periods. The failure modes shifted from ductile behavior dominated by steel yielding to more brittle modes with earlier instability [60]. Zhang et al. [69] investigated the axial behavior of square thin-walled CFST stub columns exposed to severe cold and acid rain environments. The results showed that the specimens predominantly failed through local buckling and transverse tensile fracture at the steel tube corners, while corrosion exhibited a more significant influence on structural performance degradation than freeze–thaw cycling under the investigated exposure conditions. One should bear in mind that the majority of experiments conducted in the framework of this topic made use of accelerated corrosion tests in order to achieve the effect of gradual destruction within the timeframe suitable for the laboratory. While being a very informative tool, which helps to study the influence of corrosion along with other factors, such an approach cannot accurately simulate the spatial distribution of corrosion as well as its morphological characteristics.

### 5.4. Effects of Corrosion on Structural Performance

Corrosion deterioration will gradually change the structural behavior of the CFST member by decreasing the mechanical effect of the steel tube and the steel–concrete composite interaction. With the increase in the degree of corrosion, changes in effective area, stiffness, and confinement of the steel will affect the load transfer mechanism. This will lead to a reduction in load-carrying capacity and a change in the failure modes. The magnitude of the change in structural behavior of the CFST member is dependent on the pattern, degree, and distribution of corrosion along with the corresponding degradation of the steel–concrete interaction.

#### 5.4.1. Load-Carrying Capacity Reduction

The reduction in axial load-carrying capacity of CFST members represents one of the most critical consequences of corrosion, directly affecting structural safety and serviceability. Generally, the effect of corrosion severity on the ability of the structure to carry loads is non-linear since progressive section loss, weakening of stiffness, and instability work together to bring about deterioration in the structure as corrosion worsens; however, studies conducted show that increased severity of corrosion progressively reduces the axial load-carrying capacity of the CFST structures. Comprehensive experimental studies on circular thin-walled CFST stub columns revealed that axial ultimate load-bearing capacity decreased progressively with increasing corrosion degree [2,21,70]. The degradation followed a reasonably linear relationship up to approximately 20–25% section loss, after which the rate of capacity reduction accelerated. The ultimate strength decreases approximately linearly with increasing corrosion rate and freeze–thaw cycles, independent of steel grade. However, Q235 specimens exhibit smaller strength reductions compared to Q345 counterparts [27].

**Figure 4:**
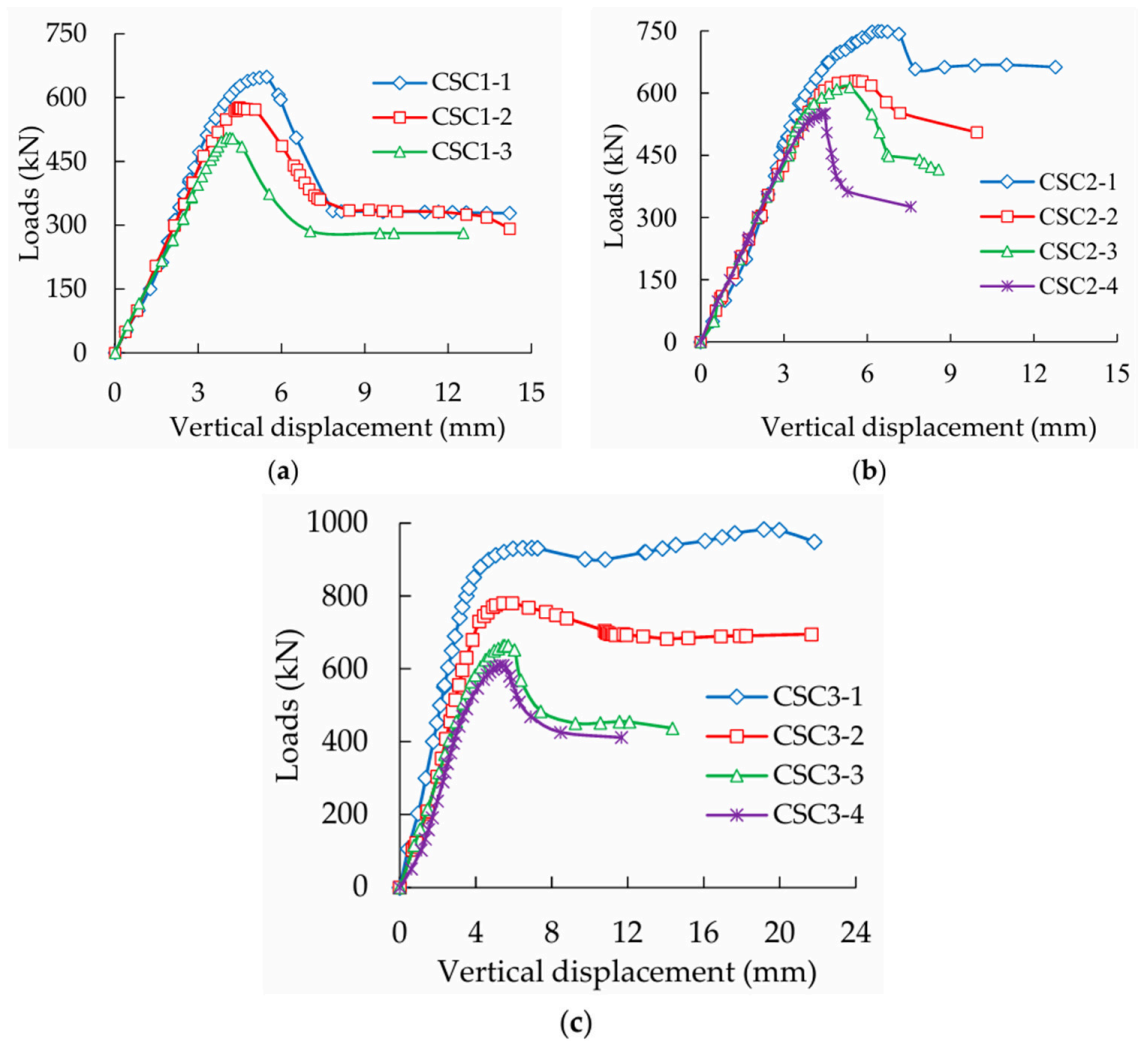
Load–vertical displacement behavior of CFST specimens with different corrosion ratios. (**a**) t = 0.92 mm; (**b**) t = 1.42 mm; (**c**) t = 1.92 mm [57].

The load–displacement response of CFST specimens (Figure 4) typically exhibits four characteristic stages: linear, nonlinear increase, steep drop, and gradual post-peak softening [55]. Initial loading is linear with no damage, followed by rust detachment and mid-height bulging. At peak load localized shear failure is initiated with a sharp load drop. Finally, load decreases gradually until complete failure.

**Figure 5:**
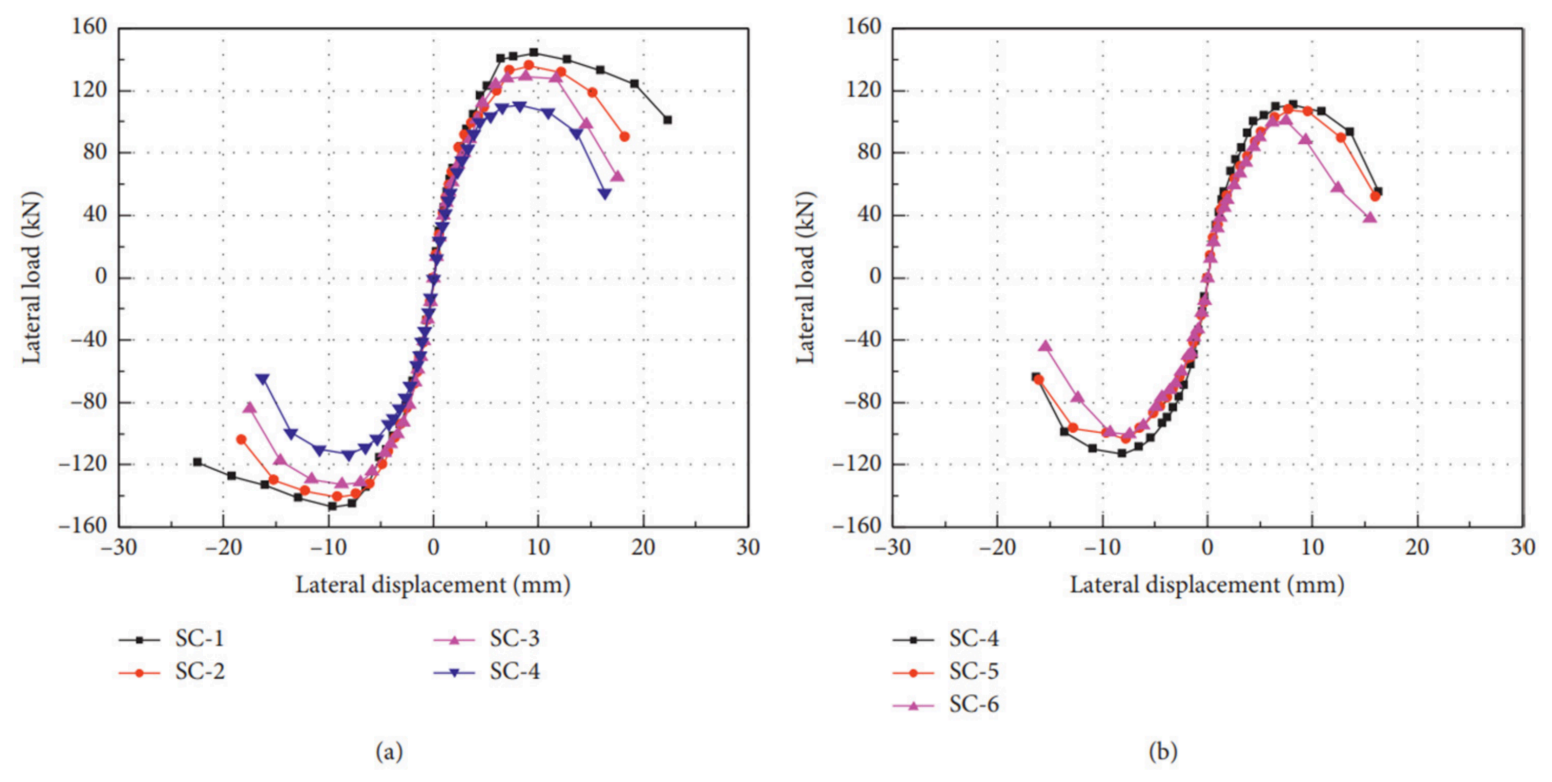
Skeleton curves of the specimens. (**a**) Corrosion-level-induced changes. (**b**) Stirrup-ratio-induced changes [70].

According to Zheng et al. [71], the skeleton curves exhibited an initial linear response, followed by nonlinear behavior after yielding and a rapid strength degradation after reaching the peak load (Figure 5). Minor asymmetry between the positive and negative loading directions was observed, which has been attributed primarily to non-uniform reinforcement corrosion, material and construction imperfections, and residual deformations accumulated during cyclic loading.

The findings demonstrate that corrosion consistently reduced the structural resistance of the members while enhancing their ductile behavior. In the case of surface corrosion caused by marine exposure alone, a resistance reduction of 11.5% was observed [58]. Xie et al. [60] reported that corrosion weakened steel plate properties and decreased CFST strength, while the use of recycled concrete aggregates had a negligible effect on flexural performance. Another study by [57] demonstrated that corrosion severity significantly reduces the compressive strength of short tubular steel columns, with the loss strongly influenced by buckling behavior. The half-wavelength of buckling governs the trend, where residual strength decreases linearly with corrosion height until it reaches a plateau once this threshold is exceeded. Additionally, increasing corrosion depth and circumferential extent consistently leads to a linear reduction in residual compressive strength. Fang et al. [63] found that the reduction in axial capacity of circular CFST columns under local corrosion is mainly due to the loss of effective steel tube area in the damaged zone. They also reported that the corrosion location has a minor effect on global behavior. Overall, the variation in load-carrying capacity remains within about 10%.

#### 5.4.2. Strain Distribution

Corrosion alters the strain distribution in CFST cross-sections through non-uniform steel thickness reduction, load redistribution, and changes in section modulus and neutral axis location. In addition, corrosion-induced material degradation and weakening of the steel–concrete interface reduce composite interaction and modify the overall structural response of CFST members.

**Figure 6:**
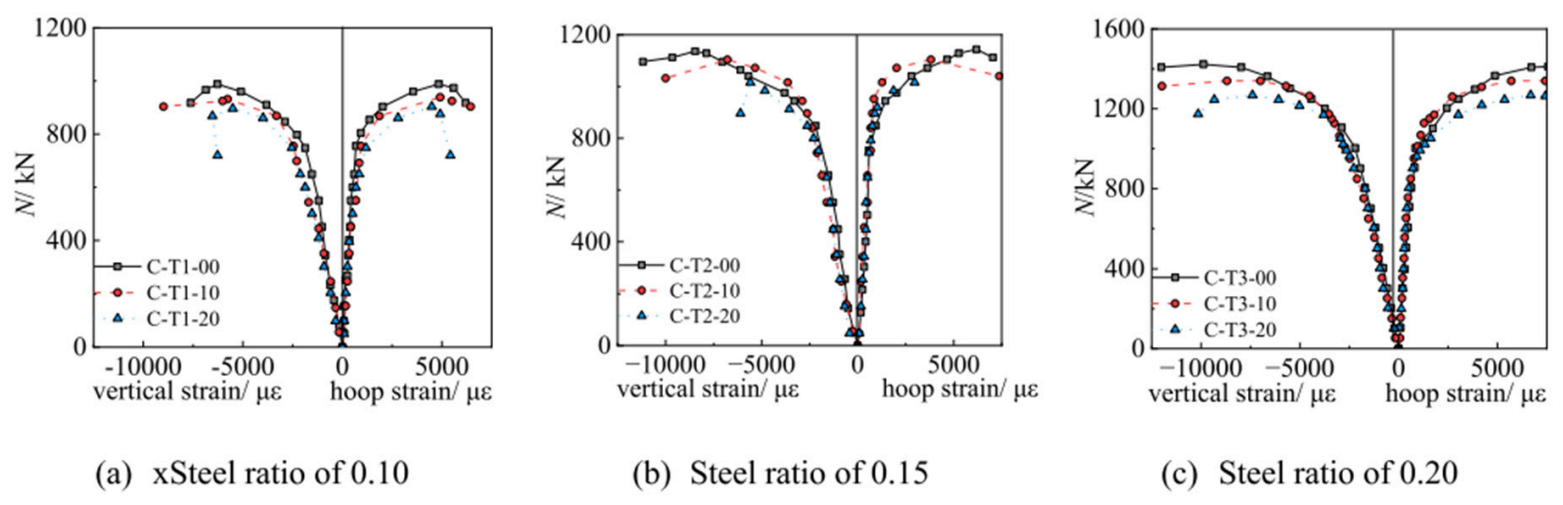
Axial load–strain curves of stub CFST columns [2].

As shown in Figure 6, the axial load–strain response shows that both vertical and hoop strains increase linearly in the elastic stage, with vertical strain developing faster than hoop strain [2]. In the elastic–plastic stage, vertical strain exceeds steel yield strain and hoop strain increases rapidly, with both strains exceeding yield at peak load. The corrosion rate has a limited influence on the overall load–strain behavior. However, higher steel ratios lead to greater strains at peak load.

The CFST columns first responded in a linear elastic manner under loading, then gradually transitioned into a nonlinear plastic stage before attaining their maximum compressive strength [72]. Fang et al. [62] observed that early load resistance is shared by the steel tube and core concrete, while stress concentration in the corroded zone leads to local bulging and eventual annular buckling. With increasing load, stress redistribution shifts the primary resistance to the core concrete. Beyond about 80% of the ultimate capacity, the response enters a pronounced elastic–plastic stage.

#### 5.4.3. Failure Modes

Corrosion-induced changes in buckling behavior represent a critical safety concern for slender CFST members and long-span structures. The reduction in wall thickness increases the diameter-to-thickness ratio, which directly elevates the slenderness of the member and reduces its buckling resistance [21,52,53,54]. Additionally, localized corrosion creates geometric imperfections and waviness that reduce the critical buckling stress according to imperfection-sensitive buckling theory.

**Figure 7:**
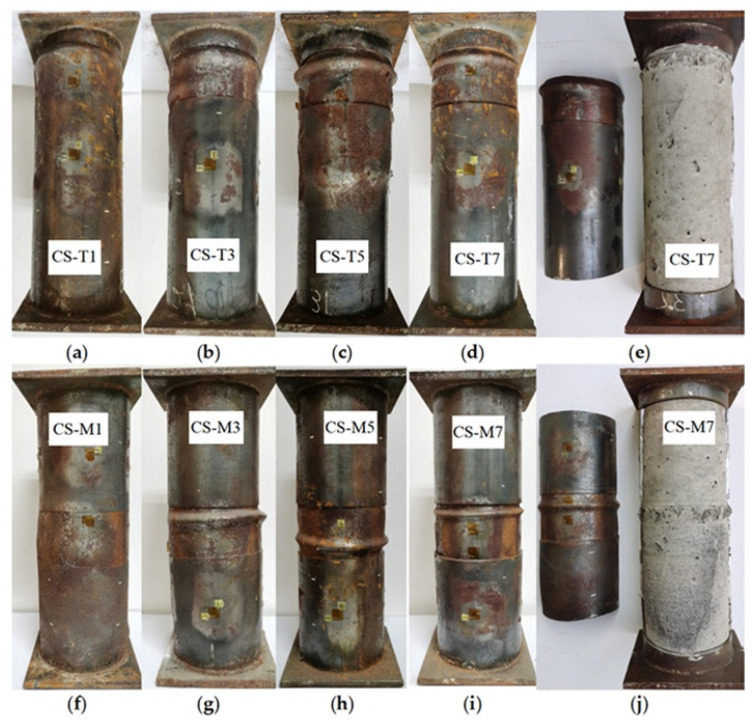
Failure patterns of CFST columns [64]: Failure modes of locally corroded CFST columns after axial compression tests: (**a**) CS-T1 (top corrosion, 10%); (**b**) CS-T3 (top corrosion, 30%); (**c**) CS-T5 (top corrosion, 50%); (**d**) CS-T7 (top corrosion, 70%); (**e**) separated steel tube and concrete core of CS-T7; (**f**) CS-M1 (mid-height corrosion, 10%); (**g**) CS-M3 (mid-height corrosion, 30%); (**h**) CS-M5 (mid-height corrosion, 50%); (**i**) CS-M7 (mid-height corrosion, 70%); (**j**) separated steel tube and concrete core of CS-M7.

Locally corroded CFST columns exhibit a consistent failure pattern characterized by local bulging in the corroded regions [63]. Micro-buckling initiates at about 50% of the ultimate load, followed by a temporary load drop and partial recovery. With increasing load, severe ring-type buckling develops, leading to final failure (Figure 7). Specimens with corrosion rates below 70% show drum-type buckling at ends and mid-height, while higher corrosion rates (>70%) result in damage concentrated mainly within the corroded zones, with relatively intact uncorroded regions.

**Figure 8:**
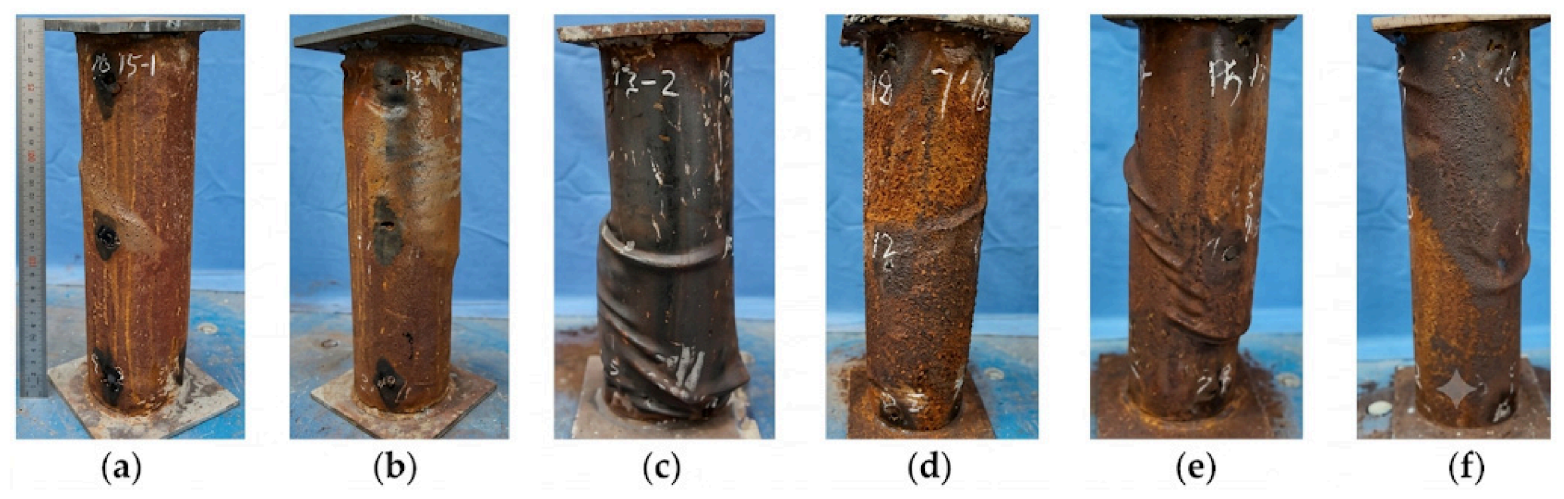
Photographs of CFST columns after failure. (**a**) CSC1-1; (**b**) CSC2-1; (**c**) CSC3-1; (**d**) CSC1-3; (**e**) CSC2-4; (**f**) CSC3-2 [54].

Photographs of failed specimens (Figure 8) show that circular thin-walled CFST columns mainly exhibit shear bulging with slight outward local buckling, regardless of corrosion condition [54]. Compared with ordinary CFST stub columns (local outward buckling) and hollow steel tubes (outward and inward buckling), the failure pattern is governed by distinct shear deformation. Shear failure planes inclined at approximately 45° are clearly observed on the steel tube surfaces, consistent with reported CFST failure modes.

**Figure 9:**
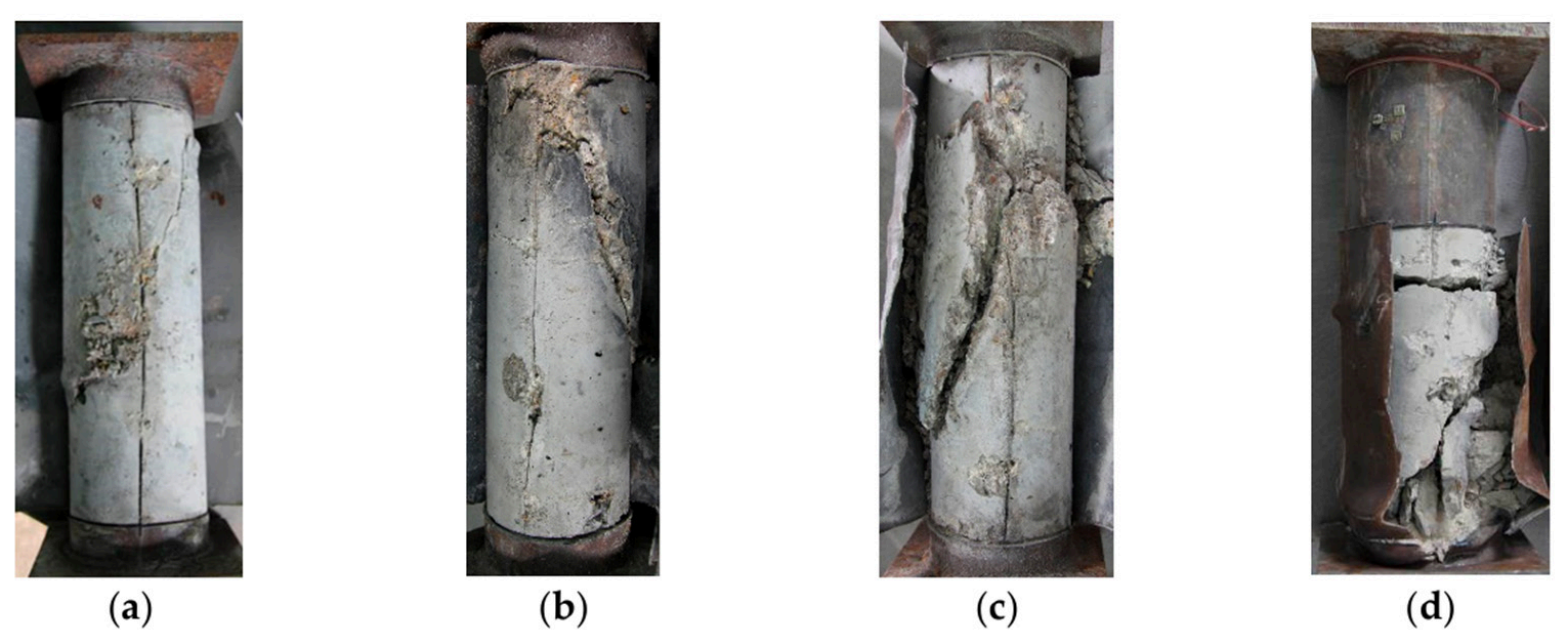
Typical photographs of the core concrete after failure. (**a**) CSC1-3; (**b**) CSC2-2; (**c**) CSC2-4; (**d**) CSC3-2 [56].

After removing the steel tubes (Figure 9), the core concrete mainly exhibited shear failure with distinct shear planes and localized crushing in CFST columns [54]. Minor initial cracks developed and propagated with loading, but were partially restrained by the steel tube confinement, eventually leading to major cracking and final concrete fracture.

Zheng et al. [71] found that differences in corrosion level and stirrup ratio had little influence on the dominant failure mode, as all six specimens failed through similar flexural-shear mechanisms under low-cycle reversed loading. Alatshan [72] examined the failure modes of the CFST columns which were predominantly governed by localized damage, with instability typically initiating in the mid-height region of the specimens where steel wall thinning occurred due to reduced thickness.

Table 3 summarizes representative experimental research work done on CFST members affected by corrosion and composite sections. These studies collectively reveal that corrosion causes degradation in terms of strength, stiffness, and capacity to carry loads. Despite the variations in the degree of degradation due to corrosion morphology, environment exposure, loading, and specimen geometry, the results obtained from these studies emphasize the significance of durability evaluation and rehabilitation of such structures.

In general, from the review of the above studies, it can be concluded that corrosion leads to deterioration in the performance of CFST structures by a combination of several deterioration processes rather than one single deterioration process. The combined effect of reduction in cross-section size of the steel, degradation in material properties, decrease in composite action, and increased vulnerability to local buckling plays a key role in deterioration.

## 6. Mitigation Strategies for CFST Corrosion

A variety of mitigation strategies have been developed to enhance the durability and service life of CFST structures exposed to corrosive environments. These approaches aim to prevent corrosion initiation, slow its progression, or minimize its impact on structural performance through material selection, protective systems, and surface treatments. The following sections review the principal corrosion mitigation techniques and their effectiveness in improving the long-term durability of CFST members.

### 6.1. Material-Based Strategies

Material-based strategies represent a fundamental approach to mitigating corrosion in CFST structures by enhancing the intrinsic resistance of both steel and concrete components against aggressive environmental conditions. Unlike external protection systems, these strategies focus on improving material properties at the design and production stages, thereby reducing the likelihood of corrosion initiation and propagation over the service life of the structure.

One of the most effective material-based approaches involves the use of corrosion-resistant steel, such as weathering steel, stainless steel, or alloyed steel with enhanced durability. Weathering steel forms a stable oxide layer that reduces further corrosion under suitable environmental conditions, while stainless steel provides superior resistance to chloride-induced corrosion due to the presence of chromium-rich passive films [78,79]. However, the high initial cost of such materials may limit their widespread application, particularly in large-scale infrastructure projects. Alternatively, the use of protective metallic coatings, such as zinc or zinc alloy layers applied during manufacturing, can significantly improve the corrosion resistance of conventional carbon steel without substantial cost increases.

The optimization of the concrete infill also plays a crucial role in corrosion mitigation. High-performance concrete (HPC) [80], ultra-high-performance concrete (UHPC) [81], and self-compacting concrete (SCC) [81,82] are increasingly employed to reduce permeability and restrict the ingress of water, oxygen, and chloride ions. The incorporation of supplementary cementitious materials (SCMs), such as fly ash, silica fume, and ground granulated blast furnace slag, enhances the microstructural density of the concrete matrix and improves its resistance to chloride penetration and carbonation [80]. These improvements help maintain a highly alkaline environment within the concrete core, which is essential for preserving the passive layer on the steel surface. Despite their advantages, material-based strategies generally provide preventive protection and have limited ability to restore structural performance once corrosion has occurred. Their effectiveness depends heavily on proper material selection, quality control during construction, and compatibility with environmental exposure conditions. Nevertheless, when implemented at the design stage, these strategies offer a cost-effective and durable solution for enhancing the long-term performance of CFST structures and are often used in combination with other protective and strengthening techniques to achieve comprehensive corrosion mitigation.

### 6.2. Protective Coatings and Surface Treatments

Protective coatings and surface treatments provide one of the most widely adopted approaches for mitigating corrosion in CFST structures by creating a barrier between the steel surface and aggressive environmental agents. Conventional systems, such as galvanization and zinc-based coatings, offer sacrificial protection against corrosion, while advanced technologies including epoxy–silica nanocomposite coatings enhance resistance through improved impermeability and durability. More recently, composite anode systems and other innovative coating technologies have been developed to combine surface protection with electrochemical corrosion control. The following subsections review these coating systems and their effectiveness in improving the long-term durability of CFST members.

#### 6.2.1. Galvanization and Zinc-Based Coating Systems

Hot-dip galvanization remains one of the most widely used and economical protective coating methods for steel reinforcement in concrete structures, providing a physical barrier against moisture and oxygen ingress [83]. However, recent research has demonstrated that alternative zinc alloy coatings can offer superior performance in highly aggressive chloride-rich environments. A comparative study of zinc-nickel (Zn-Ni) coated versus pure zinc-coated reinforcing steel in simulated concrete pore solutions revealed that electroplated Zn-Ni coatings with 13% nickel content exhibited significantly slower dissolution rates of corrosion products in chloride-rich environments compared to pure zinc coatings [84]. The Zn-Ni alloy presents a viable alternative for protecting steel in concrete structures where high chloride penetration is anticipated, combining the economic benefits of zinc coatings with enhanced durability [85].

The application of coatings on steel rebar, including epoxy and hot-dip galvanized zinc coatings, represents a critical protective strategy in the broader arsenal of corrosion mitigation methods available to engineers [83,84]. However, the effectiveness of these coatings depends significantly on their integrity during placement and their long-term adherence to the substrate under moisture and stress conditions. Regular inspection and maintenance protocols are therefore essential components of any coating-based protection strategy.

#### 6.2.2. Epoxy–Silica Nanocomposite Coatings

Advanced composite coatings combining epoxy resins with silica nanostructures have emerged as promising solutions for long-term corrosion protection. The structural properties of epoxy–silica barrier coatings are significantly influenced by the ratio of resin to curing agent, with electrochemical impedance spectroscopy studies demonstrating that intermediate DETA to DGEBA ratios of 0.4 provided the best long-term corrosion protection, achieving low-frequency impedance modulus values of up to 3.8 GΩ cm^2^ in both NaCl and simulated concrete pore solutions [85]. The superior performance of these optimized formulations is attributed to the presence of larger silica nanodomains that function as fillers within the cross-linked epoxy matrix, creating an efficient diffusion barrier against chloride ions and moisture ingress [86].

These advanced coating systems provide extended protection across different concrete aging stages, from fresh hydration (pH 14) to carbonated conditions (pH 8), making them suitable for structures experiencing varying environmental conditions throughout their service life [87]. The development and optimization of such nanocomposite coatings represent a significant advancement in protective coating technology for CFST structures and other steel–concrete composite systems.

#### 6.2.3. Composite Anode Systems and Alternative Coating Technologies

Some of the recent advancements in surface protection include the development of composite anodes, which combine the concepts of cathodic protection with those of protective coatings. Generally, cathodic protection systems can be categorized into two broad types, namely galvanic cathodic protection (GCP) and impressed current cathodic protection (ICCP). The choice between these types of cathodic protection systems is based on the nature of the structure [88,89]. The Composite Quantum Anode System, which entails the application of conductive coats on concrete, has been used successfully for the soft realkalization of steel–concrete interfaces on many occasions, including major rehabilitation works on carbonated concrete structures [90]. In addition to this, these systems offer an alternative to traditional repairs through the use of new mortar without making any changes in terms of weight and size. However, for these systems to work properly, there is a need for appropriate installation of anodes along with monitoring the system periodically [88]. The viability of such systems in practice is proved by multiple large field-scale projects conducted in different parts of Europe.

### 6.3. Polyurethane and Ceramic Coatings

Polyurethane and ceramic coatings have emerged as advanced surface protection systems for enhancing the corrosion resistance of CFST structures, particularly in aggressive environments such as marine, industrial, and chemically exposed conditions. Polyurethane coatings are widely recognized for their excellent adhesion, flexibility, and resistance to moisture and ultraviolet (UV) radiation [91,92]. These coatings form a dense, impermeable barrier that effectively limits the ingress of water, oxygen, and chloride ions, thereby reducing the likelihood of electrochemical corrosion reactions on the steel surface. In addition, their elastic nature allows them to accommodate minor deformations and thermal expansion of the steel tube without cracking, which is particularly beneficial for structures subjected to cyclic loading or temperature variations. As a result, polyurethane coatings are often applied as topcoat systems in multi-layer protective schemes to provide long-term durability and aesthetic protection [93].

Ceramic coatings, on the other hand, offer superior hardness, chemical stability, and resistance to high temperatures and abrasion [93,94]. These coatings are typically composed of inorganic compounds that form a highly dense and chemically inert layer on the steel surface. The primary advantage of ceramic coatings lies in their exceptional resistance to aggressive chemical environments, including acidic and alkaline conditions, which are commonly encountered in industrial and offshore applications. Furthermore, their low permeability significantly reduces the diffusion of corrosive agents, thereby enhancing long-term protection. However, ceramic coatings are generally more brittle than polymer-based coatings and may require careful surface preparation and controlled application techniques to prevent cracking or delamination [90].

Despite their excellent protective performance, both polyurethane and ceramic coatings have limited direct influence on the structural behavior of CFST members, as they primarily function as preventive measures rather than strengthening systems. Their effectiveness is highly dependent on coating integrity, surface preparation, and long-term maintenance. In practice, these coatings are often used in combination with other mitigation strategies, such as cathodic protection or FRP wrapping, to achieve a more comprehensive and durable corrosion protection system.

### 6.4. Internal Tube Coatings for Hollow and Pre-Filled Tubes

Internal corrosion of steel tubes in CFST members, although less visible than external corrosion, poses a significant threat to long-term durability and structural performance [95]. To address this issue, internal tube coatings have been developed as a preventive measure for both hollow steel tubes prior to concrete filling and pre-filled tubes in existing structures. These coatings are designed to protect the inner surface of the steel tube from moisture, oxygen, and aggressive chemical species that may penetrate through the concrete core or enter through micro cracks and construction defects. For hollow steel tubes, internal coatings are typically applied during the fabrication stage before concrete infilling. Epoxy-based coatings, zinc-rich primers, and other corrosion-resistant linings are commonly used due to their strong adhesion and chemical resistance. These coatings create a protective barrier that prevents direct contact between the steel surface and potentially corrosive agents within the concrete pore solution. In addition, they can improve the durability of CFST members exposed to chloride-rich environments, where internal corrosion may initiate at the steel–concrete interface [96]. From a structural perspective, internal coatings do not significantly enhance the load-carrying capacity, ductility, or buckling resistance of CFST members. Nevertheless, they play a crucial role in preserving the integrity of the steel–concrete interface, which is essential for maintaining composite action. By reducing the risk of hidden corrosion, internal coatings contribute to improved durability and service life, particularly in structures exposed to harsh environmental conditions. Consequently, their use is recommended as part of an integrated corrosion mitigation strategy, especially for critical infrastructure where long-term performance is a primary concern. For ease of implementation of the findings discussed above, Table 4 provides a brief summary on the relation between exposure conditions, main corrosive mechanisms, their effects, and recommended measures of mitigating corrosion. The aim of this summary is to help researchers and engineers choose appropriate means of combating corrosion in CFST structures depending on the prevailing condition of deterioration.

## 7. Strengthening and Retrofit Techniques

A range of strengthening and retrofit techniques has been developed to restore the structural capacity of corroded CFST members and extend their service life under aggressive environmental conditions. These techniques differ in their strengthening mechanisms, installation requirements, durability, and cost-effectiveness. The following subsections review the most widely adopted retrofit approaches, including FRP wrapping systems, fabric-reinforced cementitixous matrix (FRCM) strengthening, steel jacketing, and other emerging rehabilitation methods. Particular attention is given to their effectiveness in improving load-carrying capacity, ductility, buckling resistance, and long-term corrosion protection of deteriorated CFST structures.

### 7.1. FRP Wrapping Techniques

**Figure 10:**
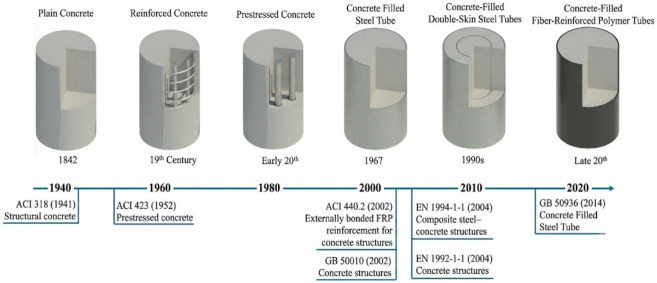
Evolution of concrete structural systems and corresponding design standards: ACI 318 [98], ACI 423 [99], ACI 440.2 [100], GB 50010 [101], EN 1994-1-1 [102], EN 1992-1-1 [103], and GB 50936 [104].

Various FRP composites, including carbon FRP (CFRP), glass FRP (GFRP), aramid FRP (AFRP), and basalt FRP (BFRP), have been widely utilized for the strengthening and retrofitting of deteriorated steel and composite structures [45,96,97]. Owing to their high strength-to-weight ratio, corrosion resistance, ease of installation, and durability, FRP systems not only enhance the structural performance of damaged members but also provide an effective protective barrier against aggressive environmental conditions, thereby mitigating further corrosion deterioration. The progression of concrete structural systems and their associated design codes is illustrated in Figure 10.

**Figure 11:**
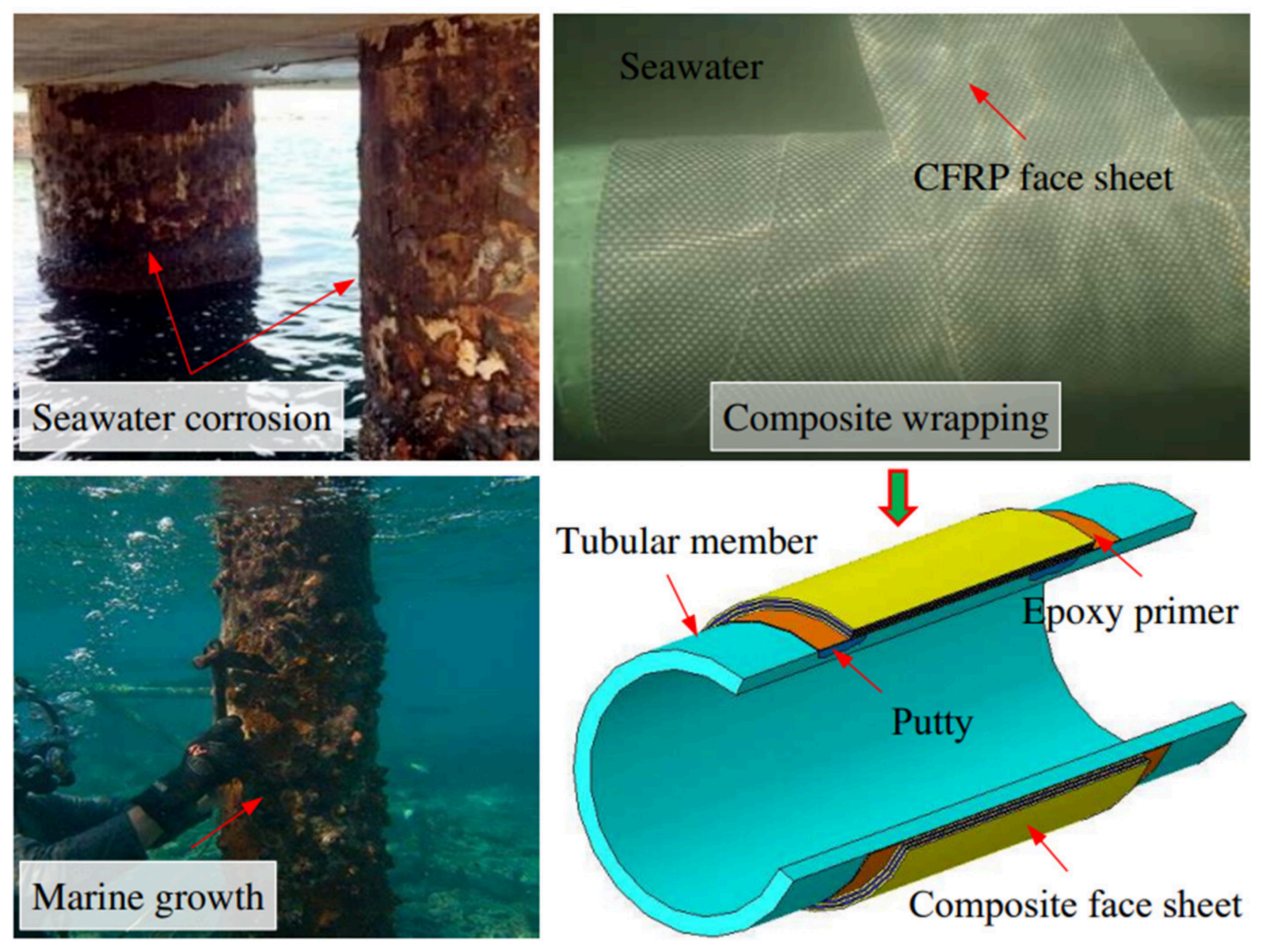
Representative diagram of the repair technique [97].

Among the available FRP strengthening techniques, externally bonded composite patch repair has emerged as an effective solution for rehabilitating corroded CFST members [20,24,28,39,97]. CFST members have recently attracted attention in structural repair and strengthening applications due to their high repair efficiency, simple installation process, and cost-effectiveness when combined with composite patches. In addition, these patches provide a protective barrier that prevents direct exposure of the steel component to seawater, thereby reducing further corrosion deterioration. Figure 11 illustrates typical corrosion-induced damage alongside a schematic of composite patch repair [105]. Compared with traditional repair techniques, the mechanical behavior of composite-strengthened CFST structures is more complex, mainly due to multiple damage mechanisms within the composite materials and the interaction between dissimilar materials. Since tubular members are primarily designed to resist compressive loads, it is particularly important to evaluate their residual compressive capacity in the presence of defects and to assess the effectiveness of composite patch repair systems.

#### 7.1.1. CFRP Wrapping

CFRP represents the most extensively studied and widely applied FRP strengthening solution for corroded CFST columns [20,82,106]. The exceptional properties of carbon fibers-including very high tensile strength (approximately 3500–4900 MPa), high elastic modulus (220–240 GPa), and superior durability make CFRP wrapping highly effective for providing lateral confinement and delaying local buckling. When applied externally to corroded CFST columns, CFRP sheets develop hoop stresses that actively confine the concrete core and restrict outward expansion of the degraded steel tube [107].

Investigations on CFRP-strengthened CFST columns have demonstrated remarkable improvements in structural performance. A comprehensive study revealed that CFRP confinement increased axial load resistance of CFST columns by 8.5% with a single sheet (1.2 mm thickness), 23.5% with double sheets (2.4 mm), 35.1% with three sheets (3.6 mm), and 44.5% with four sheets (4.8 mm) [107]. The effectiveness of CFRP wrapping is particularly pronounced in controlling local buckling of the steel tube, which typically initiates near the column ends where bending moments are highest. Beyond strength enhancement, CFRP wrapping significantly improves ductility and deformation capacity, allowing corroded columns to absorb greater inelastic deformation before reaching ultimate failure [108,109].

Regarding the application of CFRP to corroded CFST members specifically, the wrapping successfully compensates for the lost confining action caused by steel tube degradation [106,107]. Research has shown that CFRP wrapping can restore or even exceed the load-carrying capacity of pristine CFST columns, provided adequate surface preparation and proper bonding conditions are ensured. The critical concern with CFRP application to corroded surfaces is ensuring adequate adhesion; therefore, careful removal of corrosion products and surface treatment becomes essential for maximizing strengthening effectiveness [28,110,111].

#### 7.1.2. GFRP Wrapping

GFRP wrapping offers a cost-effective alternative to CFRP for strengthening corroded CFST columns, with the added advantage of lower material cost and improved resistance to alkaline concrete environments [45]. While glass fibers possess lower tensile strength (approximately 2000–3500 MPa) and significantly lower elastic modulus (70–80 GPa) compared to carbon fibers, GFRP composites demonstrate excellent durability and chemical resistance, making them particularly suitable for aggressive marine and industrial environments [28,112].

Experimental studies comparing GFRP-confined CFST specimens with unconfined controls have demonstrated consistent strength improvements. A representative study on tin slag polymer concrete wrapped with GFRP showed ultimate strength enhancements of approximately 44.5% for mono-GFRP confinement compared to unconfined specimens [45]. An important advantage of GFRP is its superior deformation capacity; GFRP-confined concrete typically exhibits greater ultimate strain and post-peak ductility compared to CFRP-confined specimens at equivalent confinement levels [113]. This characteristic makes GFRP particularly valuable for applications prioritizing seismic resilience and energy dissipation capacity.

The lower stiffness of GFRP presents both advantages and disadvantages for corroded CFST retrofit [112]. While the reduced lateral stiffness provides less dramatic initial strength enhancement, it allows for more flexible confinement that accommodates greater concrete dilation and better distributes stresses, potentially delaying rupture failure of the wrapping material. This behavior explains why GFRP-wrapped columns often demonstrate superior ductility compared to stiffer CFRP-wrapped specimens [114]. Additionally, the lower cost of GFRP materials and simpler application procedures make this technique economically attractive for large-scale retrofit projects where cost-effectiveness is a critical consideration.

#### 7.1.3. AFRP Wrapping

AFRP (commonly known as Kevlar-based FRP) offers unique performance characteristics distinct from both CFRP and GFRP, making it particularly suitable for applications requiring high ductility and energy absorption capacity [115]. Aramid fibers possess tensile strength comparable to glass fibers (approximately 3000–3150 MPa) but significantly higher strain capacity and exceptional impact resistance. The superior energy absorption capability of AFRP makes it especially valuable for strengthening corroded CFST columns in seismic regions where high deformation capacity is essential [115,116,117].

A study was conducted to assess the residual axial compressive strength of corroded circular hollow steel tubes and the effectiveness of AFRP patch repairs [105]. The analysis showed that AFRP patches can effectively restore load-carrying capacity and mitigate local buckling or regional collapse, although improvements in ductility were limited and dependent on the failure mode. Damage assessment further revealed that fiber compression failure was the predominant failure mechanism, while the mechanical properties of the putty layer played a critical role in repair performance.

#### 7.1.4. BFRP Wrapping

BFRP represents an emerging and environmentally sustainable alternative to traditional FRP composites, derived from naturally occurring basalt rock [118]. Basalt fibers demonstrate tensile strength in the range of 2000–3000 MPa with an elastic modulus of approximately 85–120 GPa, positioning them between GFRP and CFRP in terms of mechanical properties. A significant advantage of BFRP over petroleum-based carbon and aramid fibers is its lower environmental impact, lower production cost, and superior thermal resistance, making it increasingly attractive for sustainable retrofit applications [28,117].

The application of BFRP to corroded CFST columns is particularly advantageous due to basalt fibers’ inherent corrosion resistance and resistance to alkaline concrete environments [26]. A comprehensive study on the effects of coupled acid–freeze erosion revealed that BFRP-reinforced specimens showed superior performance in preserving concrete integrity compared to unconfined specimens under aggressive environmental conditions. This makes BFRP wrapping especially suitable for the retrofit of corroded columns in coastal, industrial, and freeze–thaw environments where continued environmental degradation is anticipated.

#### 7.1.5. Comparative Analysis of FRP Wrapping Methods: Confinement Benefits

The findings of this review consistently indicate that externally bonded FRP systems, particularly CFRP wraps, represent the most extensively investigated and effective approach for rehabilitating corroded CFST members. Experimental evidence from multiple studies [76,82,119,120,121] demonstrates that CFRP confinement can successfully restore the axial load-carrying capacity lost due to corrosion and, in many cases, enhance the structural performance beyond that of the original uncorroded specimens. This positive effect has been observed under various corrosion scenarios, including chloride-induced corrosion [119], regional corrosion [82], artificial defects [120], and pitting corrosion [121], highlighting the robustness of FRP confinement as a strengthening mechanism.

The key characteristics of these studies on externally bonded FRP/CFRP strengthening are summarized in Table 5, which provides a comparative overview of the experimental configurations, corrosion simulation methods, loading types, and key performance indicators.

The effectiveness of CFRP strengthening is evident across different column geometries, concrete types, and loading conditions. Although the degree of improvement varies depending on parameters such as corrosion severity, number of FRP layers, corner radius, and filler materials, the overall trend remains consistent: external FRP confinement compensates for the loss of steel confinement caused by corrosion and significantly enhances structural resistance [20,119]. These findings reinforce the fundamental role of confinement in governing the behavior of CFST members and emphasize the contribution of FRP wraps as an additional confinement source.

Despite the favorable short-term structural performance, concerns remain regarding the long-term durability of FRP-strengthened systems in aggressive environments. The study by [28] showed that FRP-retrofitted CFST columns performed satisfactorily under normal marine exposure but experienced durability degradation under severe triple-salinity conditions combined with wet–dry cycles. This suggests that the effectiveness of FRP systems may be influenced by environmental deterioration of the adhesive interface and polymer matrix. Consequently, while FRP wrapping provides an effective rehabilitation solution, its long-term performance as a corrosion protection system requires further validation, particularly in harsh marine environments.

From a practical perspective, the reviewed studies provide valuable guidance for engineering applications. The use of high-strength grout fillers, increased FRP layer thickness, and appropriate corner rounding in square sections can substantially improve strengthening effectiveness [20,119]. Furthermore, simplified design models proposed by [28,121] facilitate the estimation of ultimate strength and impact resistance of retrofitted members, supporting the incorporation of FRP strengthening techniques into routine engineering practice.

In addition to structural retrofitting, corrosion prevention strategies have also demonstrated promising results. The use of corrosion inhibitors investigated in [95] reduced corrosion-induced mass loss by more than 80% under severe cyclic exposure conditions. The combination of 5 kg/m^3^ Na_2_MoO_4_ and 15 kg/m^3^ benzotriazole was identified as particularly effective. However, these findings are currently limited to laboratory-scale investigations, and their long-term performance under realistic service conditions remains uncertain. Therefore, corrosion inhibitors should presently be regarded as complementary preventive measures rather than standalone alternatives to structural rehabilitation.

The selection of an appropriate strengthening material depends on several factors, including structural function, environmental exposure, durability requirements, cost constraints, and expected service life. Different FRP systems offer distinct advantages in terms of strength, ductility, corrosion resistance, thermal stability, and sustainability. Table 6 summarizes the recommended strengthening and retrofitting solutions for various engineering applications, highlighting the most suitable options, alternative choices, and the primary considerations governing material selection.

It is worth mentioning that some of the available information about the efficiency of FRP/FRCM is based on research conducted on reinforced concrete and steel structures. Although such information is useful, it is necessary to carry out additional experiments and tests in order to investigate the efficiency of CFRS structures in the future.

#### 7.1.6. Delaying Local Buckling in Corroded Zones

Local buckling of the steel tube represents the critical failure mode for CFST columns under compression, particularly when corroded thin-walled sections are considered [20]. The buckling of the steel tube wall reduces the bearing capacity and initiates concrete crushing due to loss of lateral support. FRP wrapping effectively delays the initiation of local buckling by providing external restraint that prevents outward tube dilation. The mechanism is particularly critical for corroded columns where steel tube wall thickness has been reduced by corrosion products [28].

A four-stage failure mode was observed in the axial compression buckling tests [28]. CFST columns strengthened with CFRP showed better buckling resistance compared to those reinforced with BFRP. Experimental investigations on CFRP-wrapped corroded CFST columns revealed that FRP confinement extends the loading range before local buckling occurs [121]. The delay in buckling manifestation directly translates to enhanced load-carrying capacity and improved deformation response. For example, CFRP wrapping of slender CFST columns has been shown to increase the critical buckling load and extend the plastic deformation phase of the load–displacement response [107]. This benefit is particularly pronounced for thin-walled tubes where local buckling would otherwise occur at relatively low stress levels.

**Figure 12:**
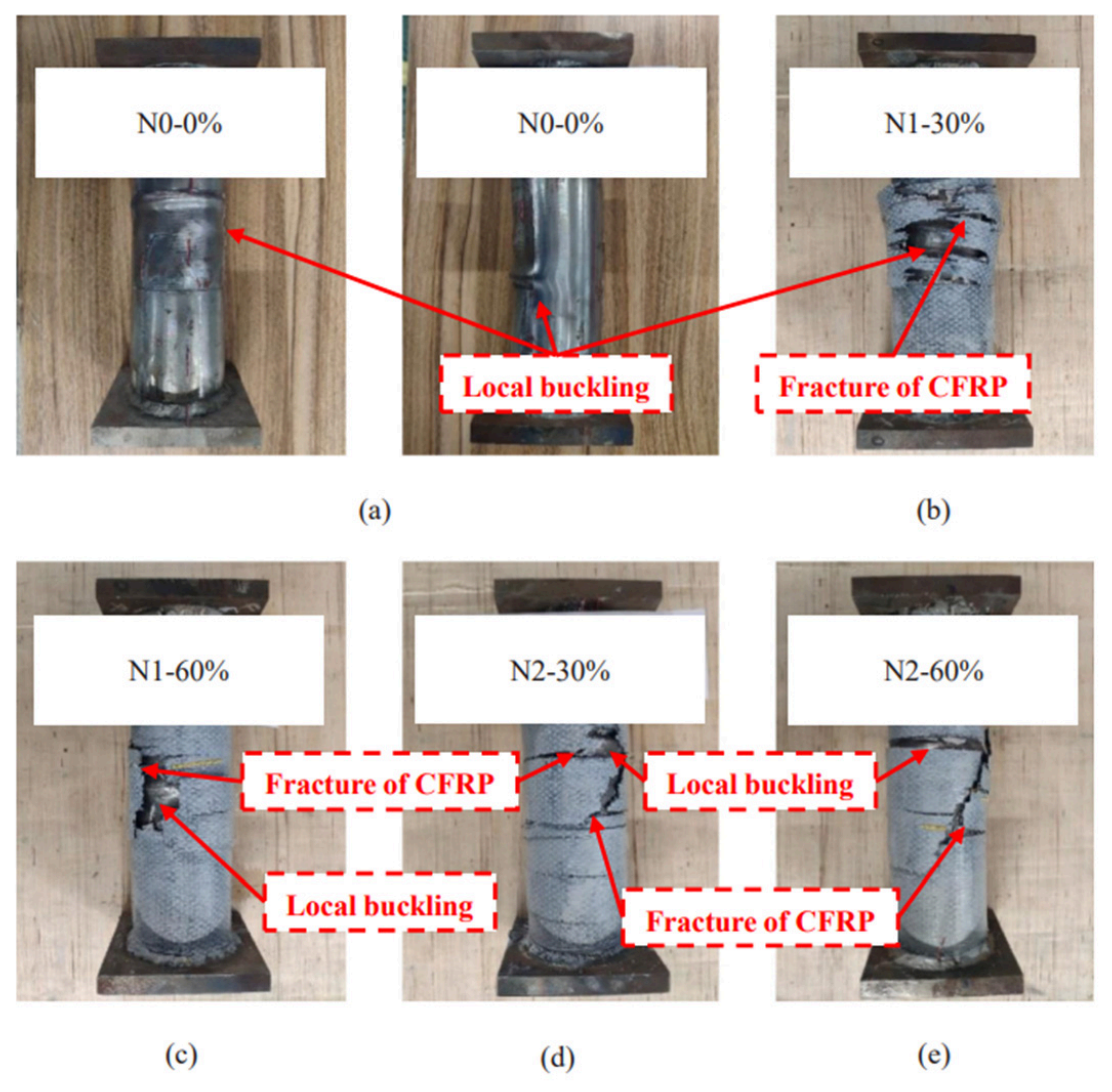
Failure modes of all test samples [105]: (**a**) N0-0%—local buckling; (**b**) N1-30%—CFRP fracture and local buckling; (**c**) N1-60%—CFRP fracture and local buckling; (**d**) N2-30%—CFRP fracture and local buckling; (**e**) N2-60%—CFRP fracture and local buckling.

Experimental and numerical studies were carried out to evaluate the axial compressive behavior of CFRP-strengthened corroded CFST stub columns subjected to preloading [107]. As shown in Figure 12, the unstrengthened corroded specimen exhibited combined compression-bending failure accompanied by severe local buckling due to corrosion-induced eccentricity. In contrast, CFRP-strengthened specimens primarily failed through CFRP rupture near the corroded region, followed by localized steel tube buckling. Different preloading levels had little influence on the observed failure mechanisms. Overall, CFRP confinement effectively reduced lateral deformation, mitigated the adverse effects of corrosion defects, and improved the stability of the corroded columns.

The effectiveness of FRP in delaying local buckling depends on both the properties of the wrapping material and the severity of corrosion in the column. Studies have quantified that local buckling can be delayed by 50–85 additional loading cycles when appropriate FRP confinement is applied, depending on the FRP type and thickness [121]. The lateral stiffness provided by FRP restrains the growth of out-of-plane deformations that would otherwise initiate buckling. Additionally, FRP wrapping helps distribute stress concentrations that typically develop at corroded zones, preventing premature localized failures. For partially corroded sections, strategic FRP placement at the most severely degraded zones can provide targeted reinforcement that maximizes the benefit-to-cost ratio [29,39]. The BFRP-strengthened specimen exhibited enhanced corrosion resistance, with minimal corrosion products observed on the outer steel tube. Minor sea sand concrete-induced deterioration was evident in the inner tube, suggesting that a single BFRP layer offers limited mitigation against sea sand concrete-related corrosion [26]. In contrast, AFRP strengthening effectively mitigates local buckling and localized collapse in corroded steel tubes, thereby enhancing their global flexural response, except under severe cross-sectional crushing. Fiber compressive rupture was identified as the dominant failure mechanism, emphasizing the primary load-transfer function of axially oriented AFRP layers. Furthermore, damage localization became more pronounced with the transition from buckling-induced failure to complete cross-sectional crushing [105].

### 7.2. FRCM Jackets

Beyond FRP-based strengthening systems, several alternative retrofit and rehabilitation techniques have been proposed to improve the structural performance and durability of corroded CFST members. These approaches employ different confinement mechanisms, material characteristics, and installation procedures to address specific structural and environmental requirements. The following subsections review the principal alternative strengthening methods, highlighting their effectiveness in restoring load-carrying capacity, enhancing ductility and buckling resistance, and providing long-term protection against further corrosion deterioration.

#### 7.2.1. Properties and Composition of FRCM Systems

FRCM (also referred to as Textile-Reinforced Mortar or TRM) represents an innovative strengthening technology that addresses specific limitations of organic polymer-based FRP composites, particularly their reduced performance at elevated temperatures and environmental incompatibility with masonry and concrete substrates. FRCM systems consist of high-strength fabric grids (typically made from carbon, glass, basalt, or PBO fibers) embedded within an inorganic cementitious matrix (cement-based mortar or engineered cementitious composite) [122,123]. The inorganic nature of the cementitious binder provides several advantages over epoxy resin matrices, including superior thermal resistance, better compatibility with concrete and masonry substrates, and enhanced resistance to UV degradation and chemical exposure.

For application to corroded CFST columns, FRCM jackets provide several distinct advantages compared to traditional FRP wrapping. First, the inorganic matrix is chemically compatible with concrete and offers superior durability in harsh environments. Second, FRCM systems are less sensitive to surface conditions and corrosion products, potentially reducing surface preparation requirements. Third, the cementitious matrix can accommodate differential movements between the strengthening system and the substrate without debonding, providing more resilient behavior [124].

#### 7.2.2. Confinement Effectiveness of FRCM Jackets

FRCM jackets applied to reinforced concrete columns demonstrate substantial improvements in confinement effectiveness and structural performance. Experimental investigations on FRCM-strengthened RC columns subjected to cyclic loading revealed significant increases in ductility, stiffness, and ultimate strength, with improvements comparable to FRP-based systems for equivalent fabric content. The lateral confining pressure developed by FRCM jackets follows similar principles to FRP wrapping, with the key difference being stress transfer through the inorganic matrix rather than direct epoxy bonding [123].

The pre-damage state of columns influences the confinement effectiveness of FRCM jackets. Investigations on pre-damaged concrete cylinders confined with PBO-FRCM revealed that prior loading history does not significantly compromise the strengthening benefits [122]. This finding is particularly relevant for corroded CFST columns that have experienced degradation; FRCM strengthening remains effective even when applied to partially damaged structures.

At present, however, direct experimental evidence on the application of FRCM systems to corroded CFST members remains limited, and further validation under representative service conditions is required.

### 7.3. Steel Jacketing Techniques

To further enhance the structural performance and durability of corroded CFST members, a range of strengthening and rehabilitation techniques have been developed beyond FRP-based systems. These methods, including steel jacketing and other advanced retrofit approaches, provide additional confinement, restore load-carrying capacity, and improve resistance to buckling and further corrosion deterioration. The following subsections examine the fundamental mechanisms, structural benefits, and practical applications of these techniques, highlighting their effectiveness in extending the service life of deteriorated CFST structures.

#### 7.3.1. Traditional Steel Jacketing Approach

Steel jacketing represents the most conventional and well-established technique for strengthening damaged reinforced concrete and composite columns [125,126]. The method involves surrounding the original column with an outer steel tube or steel plate jacket and filling the annular space with concrete or grout. This technique has been applied extensively to earthquake-damaged structures, corrosion-degraded columns, and elements requiring significant strength restoration. The fundamental principle of steel jacketing is to provide external lateral confinement through a new steel tube that supplements or replaces the confining action of the degraded original tube in CFST columns.

Steel jacketing exhibits several operational advantages for the retrofit of corroded CFST columns. First, the technique provides maximum confinement effectiveness due to the very high modulus and yield strength of steel, enabling substantial strength enhancement with reasonable material thickness. Second, steel jackets can be designed with precise thickness and dimensions to achieve targeted strength restoration. Third, the method accommodates large deformations and allows reuse of the original structure without extensive modifications. Research on slender CFST square columns strengthened with square CFST jackets demonstrated that post-strengthening columns benefit from both sectional enlargement and improved concrete confinement, resulting in substantial load-bearing capacity and ductility enhancements [125,126,127].

A practical case study of seismic retrofit in southern Italy employed steel jacketing as the primary strengthening technique for corroded RC columns [127]. The widespread adoption of steel jackets, coupled with composite floor construction, granted excellent confinement of beam-column joints and overall structural strengthening for both gravitational and seismic loads. The study confirmed that steel jacketing, when properly designed and executed, provides reliable strength restoration and improved seismic performance.

#### 7.3.2. Material Selection and Design Considerations

The selection of appropriate steel grades for jacket design significantly influences the retrofitting effectiveness and economic feasibility [127]. Research comparing different steel grades (ranging from 262 MPa to 390 MPa yield strength) revealed that increasing steel strength improves both ultimate bearing capacity and ductility of strengthened columns. When steel tube thickness increases from 5 mm to 8 mm, ultimate bearing capacity improves by 10.51–31.77%, with ductility enhancements of 6.48–17.20%. These findings establish design optimization strategies for achieving desired performance with minimal material consumption.

The thickness of sandwiched concrete between the original and outer steel tubes significantly affects confinement effectiveness. Research on axially loaded square CFST columns strengthened with circular steel tubes and sandwiched concrete jackets revealed increase in load-bearing capacity can be achieved compared to non-strengthened columns [123]. The outer steel tube’s diameter-to-thickness ratio and the strength of sandwiched concrete both influence the strengthening effectiveness; higher quality concrete and thicker outer tubes provide improved confinement and delayed local buckling [125].

#### 7.3.3. Comparison of Steel Jackets with FRP Systems

Steel jacketing and FRP wrapping represent distinctly different approaches to column strengthening, each with characteristic advantages and limitations [40]. Steel jackets provide maximum lateral stiffness and confining pressure, achieving ultimate strength enhancements of 50% or higher in optimal applications [125]. This superior strength performance makes steel jacketing the preferred choice for applications requiring maximum strength restoration or where large-diameter columns with very high confinement requirements are being retrofitted.

However, steel jackets have significant drawbacks that limit their applicability in modern retrofit practice. The increased cross-sectional dimensions from steel jacketing substantially enlarge the column footprint, creating aesthetic concerns and requiring architectural modifications to buildings [126]. This dimensional increase is particularly problematic in existing buildings where floor-to-ceiling clearances are limited or where aesthetic preservation is required [40]. Additionally, steel jackets require complex detailing of connections, welding operations, and site-based construction activities that increase labor costs and project duration [123].

Corrosion protection of steel jackets presents a long-term challenge requiring continued maintenance [128]. Unlike FRP composites which inherently resist corrosion, steel jacket surfaces must be continuously maintained through painting or coating to prevent deterioration. The environmental and economic implications of ongoing maintenance make FRP systems increasingly attractive for long-term retrofit solutions. Cost comparison studies indicate that while initial material costs of steel are lower than FRP, the lifecycle costs including fabrication, installation labor, and maintenance often favor FRP strengthening, particularly for marine and chemically aggressive environments [113].

## 8. Comparative Evaluation of Mitigation Strategies

The wide range of corrosion mitigation strategies available for CFST structures necessitates a systematic and comparative assessment to support effective decision-making in design, maintenance, and retrofit practices [20,48]. Each technique exhibits distinct advantages and limitations depending on factors such as the type of corrosion, structural performance requirements, economic considerations, and practical constraints associated with implementation. Therefore, a comprehensive evaluation framework is essential to identify the most suitable approach under varying service conditions [118,119,120,121].

In this section, the performance of commonly adopted mitigation strategies is comparatively examined based on four key aspects: (i) effectiveness against different corrosion types, including general, localized, and circumferential corrosion; (ii) influence on structural behavior, particularly in terms of residual strength, ductility, and buckling resistance; (iii) cost–benefit and lifecycle implications; and (iv) practical implement ability for both new construction and retrofitting of existing CFST members. A summarized comparison of these strategies is presented in Table 7, which highlights their relative performance across these evaluation criteria and provides a concise reference for selecting appropriate corrosion mitigation solutions.

### 8.1. Effectiveness Based on Corrosion Type

The effectiveness of corrosion mitigation strategies in CFTS structures is strongly dependent on the nature and morphology of corrosion, including general corrosion, localized corrosion, and circumferential corrosion [21,54]. General corrosion, characterized by uniform wall thinning, can be effectively controlled through material-based approaches and surface protection techniques [21]. Protective coatings such as galvanization, epoxy-based systems, and advanced nanocomposite coatings act as physical barriers that limit the ingress of oxygen, moisture, and chloride ions, thereby reducing the overall corrosion rate. In addition, cathodic protection systems provide uniform electrochemical protection across the steel surface, making them particularly effective for mitigating general corrosion in large-scale structures.

In contrast, localized corrosion, particularly pitting corrosion, presents a greater challenge due to its highly concentrated and aggressive nature [41]. This type of corrosion leads to significant stress concentrations and early crack initiation, which cannot be fully addressed by conventional coatings alone. Strengthening techniques such as FRP wrapping, especially CFRP, are effective in redistributing stresses and reducing the detrimental effects of localized defects. Furthermore, advanced nanocomposite coatings also contribute to improved resistance by enhancing microstructural impermeability against chloride ingress [106,107].

Circumferential corrosion, which often develops around the perimeter of the steel tube, significantly reduces confinement and compromises the composite interaction between steel and concrete [62]. In such cases, structural retrofit techniques become essential. Steel jacketing and FRP wrapping are particularly effective, as they restore confinement and enhance load transfer mechanisms. Similarly, FRCM systems provide uniform confinement and improved compatibility with the concrete substrate, making them suitable for repairing circumferentially corroded members [123,128].

Generally, prevention approaches like coatings and cathodic protection work well when the intention is to inhibit corrosion, while reinforcement methods like FRP, FRCM, and steel jackets have been found to be useful where there is already some deterioration of the structure.

### 8.2. Influence on Structural Behavior

Corrosion mitigation strategies not only serve to prevent further degradation but also play a crucial role in enhancing the structural performance of CFST members. One of the most significant aspects is the restoration or improvement of residual strength. Experimental studies have shown that CFRP wrapping can increase the axial load-carrying capacity of CFST columns by up to 40–45%, depending on the number of layers and confinement level [107,121]. Steel jacketing, due to its high stiffness and strength, can achieve even greater strength recovery, often exceeding 50% in well-designed applications [125].

Ductility is another critical parameter influenced by mitigation strategies. FRP systems, particularly GFRP and AFRP, are known to significantly improve ductility due to their higher strain capacity and energy absorption characteristics. FRCM systems also contribute to enhanced deformation capacity, especially under cyclic loading conditions, owing to their inorganic matrix and better compatibility with concrete. On the other hand, steel jacketing provides moderate improvements in ductility but may increase structural stiffness, potentially reducing deformation capacity in certain applications [108,113,125].

Buckling resistance is particularly sensitive to corrosion-induced wall thinning and geometric imperfections. FRP wrapping effectively delays local buckling by providing external confinement, thereby increasing the critical buckling load and extending the plastic deformation range. Steel jacketing offers substantial improvements in both local and global buckling resistance due to its high stiffness. However, internal coatings and material-based approaches have minimal direct influence on buckling behavior, as they primarily act as preventive rather than strengthening measures.

### 8.3. Cost–Benefit and Lifecycle Assessment

The selection of an appropriate corrosion mitigation strategy requires careful consideration of cost–benefit and lifecycle performance [29,39]. Initial costs vary significantly among different methods. Protective coatings generally involve low to moderate material and application costs, making them attractive for large-scale implementation. In contrast, FRP systems have relatively high material costs but benefit from rapid installation and reduced labor requirements. Steel jacketing, although associated with lower material costs, often incurs high labor and installation expenses due to welding, fabrication, and site constraints.

Maintenance costs also differ considerably. Coating systems require periodic inspection and reapplication to maintain effectiveness, leading to higher long-term maintenance demands [85,94]. Cathodic protection systems require continuous monitoring and maintenance of electrical components, including power supply and anode systems. In contrast, FRP and FRCM systems exhibit excellent durability and corrosion resistance, resulting in relatively low maintenance requirements over their service life [119,125,128].

In terms of service life extension, cathodic protection systems are among the most effective, with the potential to extend structural service life by 30–50 years under proper design and maintenance [58]. FRP wrapping and FRCM systems also provide substantial service life enhancement by improving structural performance and protecting against further degradation. Advanced coatings and material-based strategies offer moderate service life extension, depending on environmental exposure and material quality [84,113,122].

## 9. Future Research Directions

Despite significant progress in the rehabilitation of corroded CFST members, several critical challenges remain unresolved and warrant focused investigation. First, there is an urgent need for long-term field monitoring and full-scale validation studies to assess the durability and structural performance of strengthened CFST systems under realistic service conditions. Future research should move beyond short-term laboratory testing and establish comprehensive databases capturing the long-term behavior of FRP-strengthened and inhibitor-treated structures exposed to aggressive environments over decades of service life.

Second, future experimental and numerical studies should investigate the coupled effects of mechanical loading and environmental degradation mechanisms, including chloride ingress, freeze–thaw cycles, wet–dry exposure, temperature variations, and sustained loading. Since these actions occur simultaneously in practice, understanding their interaction is essential for accurately predicting deterioration rates and residual structural capacity.

Third, the existing literature is heavily concentrated on circular CFST members, while square, rectangular, and irregular composite sections remain relatively unexplored. Research should therefore focus on understanding the influence of geometry, corner stress concentrations, and confinement efficiency on corrosion progression, failure mechanisms, and retrofit effectiveness. Furthermore, strengthening strategies for ultra-high-strength CFST columns and other advanced composite structural systems require systematic investigation to support their increasing use in modern infrastructure.

Another important research priority is the development of integrated life-cycle prediction frameworks capable of linking corrosion evolution, material degradation, structural performance, and retrofit effectiveness throughout the entire service life of a structure. Such models should incorporate probabilistic deterioration mechanisms, environmental uncertainties, and maintenance interventions to facilitate performance-based design, asset management, and resilience-oriented infrastructure planning.

Comparative assessments of existing and emerging mitigation technologies are also needed. Future studies should systematically evaluate the structural effectiveness, durability, sustainability, constructability, and economic feasibility of different solutions, including FRP systems, corrosion inhibitors, protective coatings, steel jacketing, FRCM systems, and hybrid strengthening approaches. Establishing standardized testing protocols would enable meaningful comparisons and support evidence-based decision-making for practitioners.

Emerging technologies offer promising opportunities for transforming corrosion management and structural rehabilitation practices. Future research should explore the integration of smart sensing systems, digital twins, structural health monitoring networks, and Internet of Things (IoT)-enabled inspection technologies for real-time detection of corrosion damage and performance deterioration. In parallel, artificial intelligence and machine learning techniques can be employed to optimize retrofit design, predict remaining service life, and support data-driven maintenance planning. The potential of self-healing cementitious materials, nano-engineered protective coatings, and multifunctional FRP composites with sensing and self-diagnostic capabilities should also be investigated.

Finally, sustainability considerations should become a central component of future retrofit research. Life-cycle assessment (LCA), life-cycle cost analysis (LCCA), and carbon footprint evaluation should be integrated into the design and selection of rehabilitation strategies. Such an approach would enable the identification of solutions that not only restore structural performance but also minimize environmental impact and maximize long-term economic benefits. Developing sustainable, resilient, and cost-effective retrofit technologies will be essential for extending the service life of aging infrastructure and achieving global carbon reduction targets.

Overall, future research should transition from isolated laboratory-scale investigations toward integrated, life-cycle-oriented, and technology-driven approaches that simultaneously address structural performance, durability, sustainability, and resilience. Such efforts will provide the scientific foundation necessary for the development of next-generation rehabilitation strategies for corroded CFST structures.

## 10. Conclusions and Research Implications

The comprehensive review of long-term corrosion mechanisms in CFST structures reveals that corrosion represents a critical threat to structural durability and long-term performance. General corrosion causes progressive uniform wall thinning, while marine atmospheric exposure and splash zone effects create particularly aggressive conditions with corrosion rates 2–3 times higher than atmospheric zones. Localized chloride corrosion, particularly in the splash zone, generates pitting and circumferential attacks that create severe stress concentrations and geometric imperfections.

The mitigation of long-term corrosion in CFST structures requires comprehensive understanding of corrosion mechanisms, selection of appropriate protective strategies based on environmental exposure conditions and structural requirements, and implementation of long-term monitoring and maintenance protocols. The available arsenal of mitigation strategies encompasses protective coatings ranging from traditional galvanization to advanced epoxy–silica nanocomposites, electrochemical protection through impressed current or galvanic cathodic protection systems, concrete quality optimization through supplementary cementitious materials and ultra-high-performance formulations, alternative reinforcement materials including stainless steel and CFRP composites, chemical inhibitors from conventional synthetic compounds to emerging green technologies, and advanced monitoring systems enabling early detection and proactive maintenance.

Effective long-term corrosion mitigation is increasingly recognized as requiring integrated, multi-faceted approaches combining several complementary strategies rather than reliance on single protective methods. Life cycle assessment frameworks that evaluate environmental impact and economic viability alongside technical effectiveness are becoming essential tools for selecting sustainable repair and maintenance strategies. The development of advanced materials, innovative protection technologies, and data-driven assessment approaches continues to expand the available options for extending service life and ensuring continued safety, reliability, and durability of CFST structures throughout extended service periods.

While the considered mitigating measures have been proven to show satisfactory results, it would also be prudent to point out that not all the evidence presented has been obtained through experiments conducted on CFST structures. The reason being some of the tests were carried out on reinforced concrete and ordinary steel members. Thus, there remains a need for more long-term research.

Combined corrosion actions-whether with sustained loading, freeze–thaw cycling, or acid rain exposure-produce substantially accelerated degradation that exceeds the sum of individual effects. The synergistic action of freeze–thaw cycles with corrosion in cold climates can reduce residual capacity by 30–50% over a two-decade period. The effects on structural performance manifest through reductions in load-carrying capacity, altered buckling behavior with increased slenderness effects, and fundamentally changed strain distributions characterized by localized concentration factors of 2–3 times nominal values.

Future research must be directed at the field testing of these corrosion prevention measures and monitoring methods, and the formulation of sustainable materials that would serve CFST structures effectively.

## Figures and Tables

**Table 1:** Literature search strategy.

Search Category	Search Keywords
CFST	“Concrete-filled steel tube” OR CFST OR “Concrete-filled steel tubular column”
Corrosion	corrosion OR durability OR deterioration OR chloride OR carbonation OR pitting
Mitigation	mitigation OR protection OR rehabilitation OR strengthening OR repair

**Table 2:** Summary of the included studies.

Author	Type of Intervention	Comparison/Control	Outcomes Measured	Conclusions
Li et al. (2025)	Experimental investigation on bond behavior of high-strength concrete-filled steel tubes using push-out tests.	Comparison of different steel strengths, tube diameters, and tube lengths.	Bond strength, bond stress–slip relationship, strain distribution, and failure mode.	Steel tube geometry and steel strength significantly influenced bond performance. A modified prediction model showed good agreement with experimental results.
Wang et al. (2025)	Experimental investigation of chloride-corroded circular high-strength self-compacting CFST stub columns under axial compression.	Uncorroded specimens versus specimens with different corrosion levels and steel ratios.	Ultimate load, stiffness, ductility, load–displacement response, strain response, confinement effect, and residual capacity.	Chloride corrosion reduced axial capacity, stiffness, and ductility, while increasing the steel ratio partially mitigated these adverse effects.
Zhang et al. (2023)	Experimental investigation of CFRP grid-reinforced ECC-strengthened CFST columns under axial compression.	Conventional CFST columns versus CFRP grid-reinforced ECC-strengthened CFST columns.	Ultimate load capacity, axial deformation, stiffness, ductility, confinement effect, and failure mode.	CFRP grid-reinforced ECC significantly improved axial load capacity, ductility, and delayed local buckling of the steel tube.
Wang et al. (2024)	Experimental and analytical investigation of latticed CFST columns with5energy-dissipation steel shear links.	Conventional latticed CFST columns versus columns incorporating steel shear links.	Load-bearing capacity, hysteretic response, stiffness degradation, energy dissipation, and failure mechanism.	Steel shear links enhanced energy dissipation and seismic performance while maintaining satisfactory structural strength.
Zhao et al. (2025)	Numerical and analytical study on CFST-CSP composite walls subjected to combined compression and bending.	Predicted capacities compared with finite-element simulations and available experimental data.	Axial-flexural interaction, ultimate strength, failure mode, and prediction accuracy.	A simplified design model accurately predicted the load-bearing capacity of CFST-CSP composite walls.
Xiang et al. (2026)	Numerical investigation of frame structures incorporating special-shaped steel-CFST composite columns under seismic loading.	Frames with conventional columns versus frames with special-shaped steel-CFST composite columns.	Seismic response, inter-story drift, stiffness, energy dissipation, and collapse resistance.	Special-shaped steel-CFST columns improved global seismic performance and reduced structural damage.
Gan et al. (2024)	Experimental and numerical evaluation of stiffened CFST column–RC beam frames subjected to cyclic loading.	Conventional frames versus stiffened CFST column frames.	Lateral load capacity, stiffness degradation, hysteretic behavior, ductility, and failure mode.	Stiffeners effectively enhanced seismic resistance and delayed local failure of CFST columns.
Medall et al. (2024)	Numerical fire design study of steel-reinforced CFST stub columns fabricated with high-strength materials.	Different material strengths and fire exposure conditions.	Fire resistance, axial capacity, temperature distribution, and failure mechanism.	High-strength materials improved ambient strength, whereas fire resistance depended primarily on thermal degradation and confinement.
Medall et al. (2025)	Experimental and numerical investigation of slender steel-reinforced CFST columns exposed to fire.	Normal-strength and high-strength material configurations under elevated temperatures.	Axial resistance, temperature development, buckling behavior, and fire performance.	High-strength materials enhanced load capacity but required appropriate fire design considerations to prevent premature instability.
Xu et al. (2022)	Experimental investigation of CFST column-to-steel beam joints with outer annular stiffeners under cyclic loading.	Joints with and without outer annular stiffeners.	Strength, stiffness, ductility, hysteretic response, and energy dissipation.	Outer annular stiffeners significantly improved joint stiffness, strength, and seismic performance.
Yang et al. (2024)	Experimental study of four-limbed circular CFST latticed beam-columns subjected to cyclic loading.	Different loading configurations and geometric parameters.	Cyclic strength, ductility, hysteretic behavior, stiffness degradation, and failure mode.	Four-limbed circular CFST members demonstrated stable cyclic behavior and satisfactory seismic resistance.
Liang et al. (2025)	Experimental and numerical investigation of segmented composite lattice CFST columns under seismic loading.	Experimental specimens validated using finite-element simulations.	Lateral resistance, ductility, stiffness degradation, energy dissipation, and failure mechanism.	Segmented composite lattice CFST columns exhibited excellent seismic performance and reliable numerical predictability.
Lu et al. (2025)	Experimental investigation of CFST-enhanced superimposed reinforced concrete shear walls subjected to cyclic loading.	Conventional RC shear walls versus CFST-enhanced shear walls.	Lateral load capacity, stiffness degradation, ductility, energy dissipation, and failure mode.	CFST enhancement significantly improved seismic resistance, ductility, and energy dissipation while delaying structural damage.
Wang et al. (2020)	Experimental study on CFRP-confined CFST columns for marine applications subjected to combined compression, bending, and torsion.	Unconfined CFST columns versus CFRP-confined specimens.	Ultimate strength, stiffness, confinement efficiency, deformation capacity, and failure characteristics.	CFRP confinement effectively enhanced load-carrying capacity and improved durability under combined loading conditions.
Lin and Wang (2023)	Analytical investigation of offshore rock-socketed CFST piles under lateral loading.	Different pile dimensions, socket depths, and loading conditions.	Lateral displacement, bending moment, pile stiffness, and soil–structure interaction.	The proposed analytical model accurately predicted the lateral behavior of offshore CFST piles.
Yuan et al. (2020)	Experimental and theoretical investigation of CFST battened built-up bridge pier columns subjected to cyclic loading.	Experimental results compared with theoretical predictions.	Hysteretic response, ductility, stiffness degradation, energy dissipation, and failure mechanism.	The proposed theoretical model successfully predicted seismic performance, while the columns demonstrated stable cyclic behavior.
Gu et al. (2025)	Conceptual design and numerical analysis of hybrid CFST latticed bridge piers with reinforced concrete lacing systems.	Conventional bridge pier configuration versus hybrid CFST lattice system.	Structural stiffness, seismic response, ultimate capacity, and failure characteristics.	The hybrid structural system improved stiffness, seismic resistance, and overall structural efficiency.
He et al. (2025)	Experimental investigation of prefabricated beam-to-CFST column connections under cyclic loading.	Various prefabricated connection details.	Joint strength, rotational capacity, stiffness degradation, ductility, and energy dissipation.	Properly designed prefabricated connections provided excellent seismic behavior and facilitated rapid construction.
Zhao et al. (2025)	Experimental investigation of FRP-confined square high-strength CFST columns with artificial corrosion pits subjected to axial compression.	Different corrosion levels with and without FRP confinement.	Axial load capacity, stiffness, ductility, confinement efficiency, and failure mode.	FRP confinement substantially restored the strength and ductility lost due to localized corrosion damage.
Li et al. (2024)	Experimental and numerical investigation of slender CFST columns with localized pitting corrosion under axial compression.	Sound specimens versus specimens with varying pitting corrosion damage.	Ultimate load, axial stiffness, deformation behavior, buckling response, and failure mode.	Localized pitting corrosion significantly reduced load-bearing capacity and accelerated instability in slender CFST columns.
Yang et al. (2025)	Experimental investigation of lithium slag–rubber concrete-filled steel tube stub columns under axial compression.	Conventional concrete-filled steel tubes versus lithium slag–rubber concrete specimens.	Axial strength, stiffness, ductility, stress–strain response, and failure characteristics.	Lithium slag–rubber concrete demonstrated satisfactory structural performance while improving material sustainability.
Ren et al. (2025)	Experimental durability investigation of seawater and sea-sand concrete-filled BFRP–steel composite tube columns exposed to simulated marine environments.	Exposure to different marine deterioration conditions over time.	Corrosion resistance, durability, residual strength, bond behavior, and structural integrity.	The BFRP–steel composite tube system effectively protected the steel tube and maintained structural performance in aggressive marine environments.
Gao et al. (2020)	Experimental investigation of circular CFST stub columns exposed to a cold-region marine atmosphere.	Unexposed specimens versus specimens subjected to marine atmospheric corrosion.	Axial load capacity, corrosion damage, stiffness, ductility, and failure mode.	Marine atmospheric corrosion reduced structural capacity and accelerated deterioration, particularly under severe environmental exposure.
Liu et al. (2024)	Numerical investigation of FRP-CFST columns subjected to seawater corrosion and compression-induced buckling.	Different corrosion levels and FRP confinement configurations.	Buckling load, deformation, residual capacity, and failure characteristics.	Seawater corrosion significantly reduced buckling resistance, whereas FRP confinement effectively improved structural performance.
Hou and Han (2018)	Numerical life-cycle assessment of deteriorated CFST structures subjected to lateral impact after corrosion.	Various corrosion levels throughout the service life.	Impact resistance, residual strength, structural reliability, and failure probability.	Long-term corrosion markedly reduced impact resistance and shortened the service life of CFST structures.
Zhou et al. (2023)	Machine learning-based strength prediction of circular CFST columns subjected to sustained axial loading and localized corrosion.	Proposed active-learning model versus conventional prediction methods.	Prediction accuracy, ultimate strength, computational efficiency, and model reliability.	The active-learning framework accurately predicted the residual strength of corroded CFST columns with fewer training samples.
Saini and Shafei (2019)	Numerical investigation of CFST bridge columns subjected to vehicle collision.	Different impact velocities, vehicle masses, and column configurations.	Impact force, lateral displacement, residual capacity, and damage patterns.	CFST bridge columns exhibited superior impact resistance, although severe damage developed under high-energy collisions.
Nguyen et al. (2022)	Experimental and numerical investigation of eccentrically loaded high-strength CFST columns with slender steel sections.	Different steel strengths, slenderness ratios, and loading eccentricities.	Ultimate load, axial deformation, buckling behavior, and failure mode.	High-strength materials improved load capacity, while slenderness and eccentricity governed structural stability.
Li et al. (2021)	Life-cycle analytical assessment of offshore FRP-strengthened CFST columns affected by steel corrosion.	Unstrengthened versus FRP-strengthened offshore CFST columns.	Service life, corrosion progression, maintenance requirements, and life-cycle cost.	FRP strengthening significantly extended service life and improved long-term structural performance in offshore environments.
Kamil et al. (2018)	Numerical investigation of local buckling in CFST columns exposed to elevated temperatures.	Various temperature levels and steel plate slenderness ratios.	Local buckling behavior, axial capacity, and fire performance.	Elevated temperatures accelerated local buckling and reduced axial resistance, particularly in slender steel tubes.
Nguyen et al. (2021)	Experimental investigation of high-strength CFST columns with slender sections under axial compression.	Different steel grades, concrete strengths, and slenderness ratios.	Ultimate strength, stiffness, deformation capacity, and failure characteristics.	High-strength materials enhanced axial resistance, while slender sections remained susceptible to global buckling.
Lee et al. (2011)	Experimental investigation of high-strength circular CFST columns subjected to eccentric loading.	Different loading eccentricities and material strengths.	Load–deflection response, ultimate capacity, stiffness, and failure mode.	Increasing eccentricity reduced axial capacity, whereas high-strength materials improved structural resistance and ductility.
Toledo et al. (2020)	Experimental investigation of short tubular steel columns with artificially fabricated local corrosion damage under axial compression.	Intact specimens versus specimens with different localized corrosion depths.	Residual compressive strength, stiffness, deformation capacity, and failure mode.	Localized corrosion significantly reduced compressive resistance, and the reduction increased with corrosion severity.
Zhang et al. (2020)	Experimental investigation of circular thin-walled CFST stub columns after corrosion.	Uncorroded specimens versus specimens with different corrosion levels.	Axial ultimate load, stiffness, deformation capacity, and failure characteristics.	Corrosion caused a progressive reduction in axial load-bearing capacity and accelerated local buckling of the steel tube.
Saini and Shafei (2018)	Numerical vulnerability assessment of CFST bridge columns subjected to combined corrosion and vehicle impact.	Columns exposed to corrosion only versus combined corrosion and impact loading.	Residual impact resistance, structural damage, energy absorption, and failure mode.	Corrosion substantially increased the vulnerability of CFST bridge columns under extreme impact events.
Alatshan et al. (2024)	Experimental investigation of locally corroded circular CFST stub columns under axial compression.	Different levels of localized corrosion compared with intact specimens.	Residual compressive strength, axial deformation, stiffness, and failure mode.	Local corrosion significantly reduced axial strength and stiffness, confirming the detrimental effect of pitting damage on CFST performance.
Xie et al. (2019)	Experimental and numerical investigation of CFST members subjected to pure bending after acid rain exposure.	Specimens exposed to acid rain versus unexposed specimens.	Flexural capacity, stiffness, crack development, and failure mode.	Acid rain deterioration reduced flexural performance and accelerated degradation of composite action.
Fang et al. (2024)	Experimental and numerical investigation of axially loaded CFST columns with localized corrosion.	Experimental results compared with finite-element predictions for different corrosion levels.	Ultimate bearing capacity, load–displacement response, stress distribution, and failure mechanism.	Localized corrosion reduced axial resistance, while the proposed numerical model accurately predicted the structural response.
Hua et al. (2019)	Finite-element investigation of square CFST beam-columns subjected to sustained loading and corrosion.	Different corrosion levels combined with long-term sustained loading.	Ultimate capacity, load-deflection behavior, stress distribution, and failure mode.	Sustained loading accelerated the adverse effects of corrosion, resulting in lower residual structural capacity.
Hou et al. (2016)	Experimental investigation of circular CFST members subjected to sustained loading and chloride corrosion.	Various chloride corrosion durations and sustained loading conditions.	Flexural behavior, load-deflection response, stiffness, and residual capacity.	Chloride corrosion combined with sustained loading significantly reduced flexural performance and structural durability.
Gao et al. (2020)	Experimental investigation of circular thin-walled CFST stub columns exposed to harsh offshore environments.	Different marine exposure durations compared with unexposed specimens.	Corrosion degree, residual strength, stiffness, ductility, and failure characteristics.	Long-term offshore exposure accelerated corrosion deterioration and reduced the residual axial capacity of CFST columns.
He et al. (2024)	Experimental investigation of square CFST stub columns subjected to freeze–thaw cycles under axial compression.	Different numbers of freeze–thaw cycles compared with intact specimens.	Ultimate load capacity, stiffness, ductility, deformation behavior, and failure mode.	Freeze–thaw damage progressively reduced compressive performance and accelerated structural deterioration of CFST columns.
Lyu et al. (2023)	Experimental investigation of circular CFST stub columns subjected to combined corrosion and freeze–thaw cycles.	Different corrosion levels and freeze–thaw cycles compared with intact specimens.	Axial load capacity, stiffness, ductility, failure mode, and residual strength.	The combined action of corrosion and freeze–thaw cycles accelerated structural deterioration and produced greater strength loss than either condition alone.
Zhang et al. (2020)	Experimental investigation of square CFST stub columns exposed to severe cold and acid rain environments.	Environmental deterioration conditions compared with unexposed specimens.	Axial compressive strength, stiffness, deformation capacity, and failure characteristics.	Combined environmental actions significantly reduced compressive performance and increased material degradation.
Yuan et al. (2022)	Experimental investigation of square CFST columns subjected to cyclic loading after acid rain exposure.	Acid rain-damaged specimens versus undamaged specimens.	Cyclic response, stiffness degradation, ductility, energy dissipation, and failure mode.	Acid rain exposure reduced cyclic performance and accelerated deterioration under repeated loading.
Han et al. (2017)	Experimental investigation of circular CFST members subjected to combined tensile loading and chloride corrosion.	Different chloride corrosion durations under sustained tensile loading.	Tensile behavior, stiffness, deformation, corrosion effects, and failure mode.	Chloride corrosion significantly reduced tensile resistance and accelerated deterioration during sustained loading.
Tang et al. (2024)	Experimental investigation of corroded CFST members with spherical cap gaps under long-term tensile loading.	Various corrosion levels and sustained tensile loading conditions.	Tensile behavior, deformation, crack development, and failure characteristics.	Long-term tensile loading combined with corrosion accelerated structural degradation and reduced residual capacity.
Nie et al. (2024)	Finite-element investigation of corroded steel-reinforced concrete columns subjected to axial compression.	Various corrosion damage levels compared with intact columns.	Axial load capacity, stress distribution, deformation, and failure mechanism.	Corrosion substantially reduced compressive resistance, and the numerical model accurately predicted the deterioration process.
Dong et al. (2020)	Experimental investigation of corrosion characteristics of concrete-filled CFRP-steel tube piles under hygrothermal environments.	Different temperature and humidity exposure conditions.	Corrosion development, durability, bond behavior, and residual structural performance.	CFRP-steel composite tubes effectively improved corrosion resistance under aggressive environmental conditions.
Khodabandeh et al. (2024)	Experimental investigation of ultra-high-performance concrete coatings for chloride-induced corrosion protection.	Uncoated reinforced concrete specimens versus UHPC-coated specimens.	Chloride penetration resistance, corrosion protection efficiency, durability, and cracking behavior.	UHPC coatings significantly enhanced resistance to chloride ingress and delayed corrosion initiation.
Atiyah et al. (2025)	Experimental investigation of CFRP-strengthened self-compacting CFST columns with simulated regional corrosion.	Corroded specimens with and without CFRP strengthening.	Strength recovery, axial capacity, stiffness, deformation, and failure mode.	CFRP sheets effectively restored structural capacity and delayed local buckling caused by corrosion damage.
Wang et al. (2024)	Experimental investigation of corrosion resistance of CFST members using a Na_2_MoO_4_–benzotriazole inhibitor system.	Conventional CFST specimens versus inhibitor-treated specimens.	Corrosion rate, electrochemical performance, durability, and protection efficiency.	The inhibitor system significantly improved corrosion resistance and enhanced the long-term durability of CFST members.
Huang et al. (2023)	Experimental and numerical investigation of locally corroded circular CFST stub columns strengthened with CFRP.	Corroded CFST columns with and without CFRP strengthening.	Ultimate load capacity, stiffness, ductility, strain distribution, and failure mode.	CFRP strengthening effectively restored axial capacity, delayed local buckling, and improved the ductility of corroded CFST columns.
Huang et al. (2024)	Experimental investigation of CFRP-strengthened circular CFST stub columns considering preload and corrosion effects.	Corroded specimens with different preload levels before CFRP strengthening.	Axial compressive strength, stiffness, ductility, confinement efficiency, and failure mechanism.	CFRP strengthening significantly enhanced residual capacity even in preloaded corroded specimens, although excessive preload reduced strengthening efficiency.
Sabih et al. (2024)	Numerical investigation of CFRP-strengthened CFST composite columns subjected to axial compression.	Unstrengthened versus CFRP-strengthened CFST columns with different CFRP configurations.	Ultimate load, stress distribution, deformation behavior, and confinement effect.	CFRP wrapping improved axial resistance and delayed local instability, with performance increasing as CFRP thickness increased.
Cai et al. (2024)	Experimental investigation of bonding behavior between CFRP patches and corrosion-damaged steel using different surface preparation techniques.	Various surface preparation methods before CFRP bonding.	Bond strength, failure mode, interfacial behavior, and durability.	Appropriate surface preparation substantially enhanced CFRP bond performance and improved strengthening effectiveness for corroded steel members.
Reddy and Sivasankar (2020)	Experimental investigation of corroded CFST columns wrapped with GFRP sheets under axial compression.	Corroded CFST columns with and without GFRP confinement.	Axial load capacity, stiffness, ductility, deformation, and failure characteristics.	GFRP wrapping effectively recovered strength and ductility while reducing the adverse effects of corrosion damage.
Dinesh and Amritha (2024)	Analytical investigation of localized corrosion damage and strengthening strategies for orthogonal CFST columns.	Different corrosion scenarios and strengthening techniques.	Residual strength, stress distribution, deformation, and rehabilitation efficiency.	Appropriate strengthening techniques effectively restored structural performance and extended the service life of corroded CFST columns.
Abdelkarim and ElGawady (2014)	Analytical and finite-element investigation of FRP–concrete–steel double-skin tubular columns.	Finite-element predictions compared with analytical models and available experimental data.	Axial capacity, confinement effect, stress distribution, and failure mechanism.	The analytical model accurately predicted structural behavior, demonstrating the effectiveness of FRP confinement in composite tubular columns.
Zhang et al. (2025)	Machine learning-based prediction of the capacity of CFST columns with damaged BFRP jackets.	Proposed machine-learning model versus conventional prediction approaches.	Prediction accuracy, residual load capacity, model robustness, and computational efficiency.	The machine-learning model provided accurate and reliable predictions of the residual capacity of damaged BFRP-confined CFST columns.
Alatshan et al. (2026)	Experimental investigation of locally corroded circular CFST stub columns retrofitted using CFRP or grout materials.	CFRP strengthening versus grout repair versus unstrengthened corroded specimens.	Residual compressive strength, stiffness, ductility, deformation, and failure mode.	Both repair techniques improved structural performance, while CFRP retrofitting generally achieved the greatest enhancement in residual load-carrying capacity.
Lang (2019)	Investigation of corrosion rate control methods for coastal highway bridge CFST structures.	Conventional protection practices versus proposed corrosion control strategy.	Corrosion rate, durability, maintenance requirements, and structural service life.	The proposed corrosion control method effectively reduced corrosion progression and extended the service life of coastal CFST bridge structures.
Liao et al. (2025)	Experimental investigation of CFRP-strengthened pitting-corroded CFST columns subjected to lateral impact loading.	Corroded specimens with and without CFRP strengthening under different impact energies.	Impact resistance, lateral displacement, energy absorption, residual capacity, and failure mode.	CFRP strengthening significantly enhanced impact resistance and reduced permanent deformation of pitting-corroded CFST columns.
Ma et al. (2025)	Experimental investigation of square CFST columns strengthened with circular steel tubes and sandwiched concrete jackets under axial compression.	Original CFST columns versus strengthened composite columns.	Axial load capacity, stiffness, confinement efficiency, deformation, and failure characteristics.	Composite jacketing substantially increased axial strength and stiffness while delaying local buckling.
Lai and Ho (2015)	Experimental investigation of thin-walled CFST columns strengthened with external circular steel jackets.	Strengthened specimens compared with unstrengthened thin-walled CFST columns.	Axial compressive strength, confinement effect, deformation capacity, and failure mode.	External steel jackets markedly improved confinement and increased the axial load-bearing capacity of thin-walled CFST columns.
Wu et al. (2026)	Experimental and finite-element investigation of square FRRC-CFST columns under seismic loading with multi-scale monitoring.	Experimental specimens validated using numerical simulations and monitoring techniques.	Seismic behavior, hysteretic response, stiffness degradation, ductility, and structural damage.	FRRC-CFST columns demonstrated excellent seismic performance, while the monitoring system effectively captured structural damage evolution.
Chen et al. (2025)	Experimental investigation of seawater sea-sand reinforced concrete composite beams confined with C-FRCM under lateral impact loading.	Conventional beams versus C-FRCM-confined composite beams.	Impact resistance, deformation, energy absorption, cracking behavior, and failure mode.	C-FRCM confinement significantly enhanced impact performance and reduced structural damage.
Feng et al. (2021)	Experimental investigation of corroded circular reinforced concrete columns strengthened with C-FRCM under cyclic loading.	Corroded columns before and after C-FRCM strengthening.	Cyclic strength, stiffness degradation, ductility, energy dissipation, and failure mode.	C-FRCM strengthening effectively restored seismic performance and improved the cyclic response of corroded columns.
Granata (2024)	Case study of seismic retrofit strategies for corrosion-damaged reinforced concrete buildings.	Existing corroded structure versus retrofitted structural system.	Structural capacity, seismic safety, rehabilitation effectiveness, and serviceability.	Appropriate retrofit strategies successfully restored structural safety and significantly improved seismic performance.
Trapko and Musiał (2020)	Experimental investigation of eccentrically compressed reinforced concrete columns strengthened with PBO-FRCM.	Unstrengthened columns versus PBO-FRCM strengthened columns.	Structural stiffness, load capacity, deformation behavior, and failure mode.	PBO-FRCM reinforcement effectively enhanced stiffness and improved the structural response under eccentric compression.
Harilal et al. (2019)	Experimental investigation of high-performance green concrete incorporating fly ash, nanoparticles, and corrosion inhibitors.	Conventional concrete versus mixtures containing fly ash, nanoparticles, and corrosion inhibitors.	Compressive strength, chloride penetration resistance, durability, and corrosion performance.	The combined use of supplementary cementitious materials and corrosion inhibitors significantly improved durability and resistance to chloride-induced corrosion.
Pokorný et al. (2024)	Experimental investigation of hot-dip galvanized prestressing reinforcement embedded in concrete.	Galvanized reinforcement versus conventional reinforcement.	Bond strength, corrosion protection efficiency, and durability.	Hot-dip galvanizing provided effective corrosion protection while maintaining satisfactory bond performance with concrete.
Bučko et al. (2024)	Electrochemical investigation of Zn-Ni-coated reinforcing steel in simulated concrete pore solutions.	Zn–Ni-coated reinforcement versus uncoated reinforcement.	Corrosion potential, corrosion current density, coating stability, and protection efficiency.	Zn–Ni coatings significantly enhanced corrosion resistance in alkaline concrete environments.
Chen et al. (2025)	Axial compression tests on UHTCC-encased steel tubular columns.	Conventional steel tubular columns versus UHTCC-encased columns.	Axial load capacity, stiffness, ductility, and failure mode.	UHTCC encasement improved axial strength, ductility, and structural performance.
Uvida et al. (2022)	Experimental investigation of epoxy-silica barrier coatings for reinforcing steel.	Uncoated reinforcing steel versus epoxy–silica-coated reinforcement.	Coating structure, corrosion resistance, adhesion, and durability.	Epoxy–silica coatings formed an effective protective barrier that significantly reduced steel corrosion.
Wang et al. (2023)	Experimental investigation of dual-filler epoxy-coated reinforcing steel under corrosive environments.	Conventional epoxy coating versus dual-filler epoxy coating.	Mechanical properties, corrosion resistance, coating durability, and adhesion.	Dual-filler epoxy coatings enhanced both corrosion resistance and mechanical performance compared with conventional coatings.
Ghamarpoor et al. (2023)	Experimental investigation of nano-silica-modified epoxy adhesive for concrete–steel bonding.	Conventional epoxy adhesive versus nano-silica-modified epoxy adhesive.	Bond strength, mechanical properties, adhesion, and durability.	Nano-silica modification substantially improved adhesive strength and bond performance between steel and concrete.
Bogatu et al. (2025)	Experimental evaluation of protective coating systems for steel exposed to marine environments.	Different protective coating systems under identical marine exposure conditions.	Corrosion resistance, coating degradation, durability, and protection efficiency.	High-performance coating systems effectively delayed corrosion and prolonged the service life of steel structures in marine environments.
Sikora and Ostrowski (2025)	State-of-the-art evaluation of external confinement methods for strengthening concrete columns.	Comparison of different external confinement techniques reported in the literature.	Structural strengthening efficiency, load capacity, ductility, and durability.	External confinement systems substantially enhanced structural capacity, particularly FRP-based strengthening methods.
Cai et al. (2024)	Experimental investigation of CFRP patch bonding to corrosion-damaged steel with different surface preparation techniques.	Different steel surface preparation methods before CFRP application.	Bond strength, interfacial behavior, failure mode, and durability.	Proper surface preparation significantly improved CFRP bonding performance and strengthening effectiveness.
Guo et al. (2024)	Experimental investigation of carbon nanotube-reinforced waterborne epoxy zinc-rich coatings for steel structures.	Conventional zinc-rich epoxy coatings versus CNT-reinforced coatings.	Corrosion resistance, coating adhesion, electrochemical performance, and durability.	Carbon nanotube reinforcement significantly improved barrier properties and prolonged coating service life.
Alam et al. (2017)	Experimental and numerical investigation of FRP-strengthened CFST members under lateral impact loading.	Conventional CFST members versus FRP-strengthened members.	Impact resistance, absorbed energy, permanent deformation, and failure characteristics.	FRP strengthening substantially increased impact resistance and reduced permanent structural damage.
Wang et al. (2024)	Experimental investigation of corrosion-resistant CFST members incorporating Na_2_MoO_4_ and benzotriazole inhibitors.	Untreated CFST specimens versus inhibitor-treated specimens.	Corrosion resistance, electrochemical behavior, durability, and protection efficiency.	The inhibitor system significantly reduced corrosion activity and enhanced long-term durability of CFST members.
Huang et al. (2023)	Experimental and numerical investigation of CFRP-retrofitted locally corroded circular CFST stub columns.	Corroded columns with and without CFRP strengthening.	Residual axial capacity, stiffness, strain development, confinement efficiency, and failure mode.	CFRP retrofitting effectively restored compressive strength and delayed local buckling in corrode.

**Table 3:** Comparison table of key corrosion studies.

Ref.	Specimen Type	Loading Condition	Corrosion Method	Key Findings	Performance Reduction
Zhang et al. [54]	Thin-walled circular CFST stub columns	Axial compression	Electrical accelerated corrosion	Failure mode shift from ductile shear-bulging to brittle patterns; linear capacity decreases with corrosion depth	Capacity decreased with increasing corrosion degree
Xie et al. [61]	Circular and square CFST beams	Flexural bending	Simulated acid rain environment	Deterioration in yield strength and elastic modulus; ultimate moment capacity decreased	15–40% reduction in ultimate strength
Zhang et al. [73]	CFST stub columns	Axial compression	Combined freeze–thaw cycles + acid rain	Material strength and steel ratio are critical parameters; design formulae proposed for residual strength prediction	30–50% reduction after 20 cycles
Zheng et al. [71]	RC columns	Reversed cyclic loading	Simulated acid rain environment	Higher corrosion degree strengthened concrete restraint; improved seismic performance	Performance degradation reduced by higher stirrup ratio
Han et al. [74]	Circular CFST tensile members	Sustained tension + chloride exposure	Electrical accelerated (120 days)	Time-dependent strength reduction; interface degradation between steel and concrete	Progressive degradation of ultimate capacity
Tang et al. [75]	Circular CFST with spherical cap gaps	Sustained axial tension + chloride corrosion	Long-term salt spray	Reduced stiffness; diminished load transfer; internal force redistribution	Significant reduction in flexibility
Toledo et al. [57]	Short tubular steel columns	Axial compression	Artificially fabricated local corrosion damage	Residual strength decreased linearly with corrosion depth/height; constant after exceeding half-wavelength buckling	Variable severity depending on damage geometry
Nie et al. [76]	SRC middle-length columns	Axial compression	Progressive rust development	Bond-slip relationship affected; residual strength reduced significantly when rust rate exceeded 1.5%	Rapid decline above 1.5% rust rate
Dong et al. [77]	CFRP-steel tube piles	Environmental exposure	Hydrothermal environment (high temperature + humidity)	Mechanical properties increased with external CFRP protection; effective corrosion protection mechanism demonstrated	Partial recovery with protective measures

**Table 4:** Relationship between exposure conditions, corrosion mechanisms, damage consequences, and recommended mitigation strategies for CFST structures.

Exposure Condition	Dominant Corrosion Mechanism	Main Consequence	Recommended Mitigation Strategy
Marine environment	Chloride-induced corrosion and pitting	Section loss and reduced load-carrying capacity	Protective coatings, cathodic protection, corrosion-resistant steel
Industrial atmosphere	General corrosion and acid attack	Uniform wall thinning	High-performance coating systems, regular maintenance
Coastal splash zone	Localized pitting and circumferential corrosion	Local buckling and stress concentration	Duplex coating systems, cathodic protection, periodic inspection
Freeze–thaw with chlorides	Combined physical and electrochemical deterioration	Concrete cracking and accelerated corrosion	Low-permeability concrete, surface sealers, crack repair
Existing corroded CFST members	Advanced corrosion damage	Reduced structural capacity	Steel jacketing, FRP/FRCM strengthening, localized repair

**Table 5:** Summary of studies on externally bonded FRP strengthening.

Ref	FRP Application Configuration	Corrosion Simulation Method	Loading Type	Key Performance Indicator
[20]	CFRP and hybrid CFRP/GFRP wraps	Artificial corrosion pits by laser cutting	Axial compression	Load-bearing capacity and ductility
[28]	CFRP and BFRP wraps	Seawater corrosion with wet–dry cycles	Axial compression	Buckling resistance performance
[77]	CFRP sheets bonded to steel tube	Hydrothermal environment simulation	Corrosion exposure	Mechanical properties and corrosion resistance
[105]	AFRP	Simulated marine corrosion environment with stochastic pits	Axial compression	Mechanical responses
[108]	CFRP sheets	Simulated regional corrosion	Axial loading	Load-carrying capacity
[119]	CFRP sheets with epoxy-putty or high strength grout	Chloride-induced localized corrosion at mid-height	Axial static compression	Ultimate compressive capacity/strength recovery
[120]	CFRP wrap	Artificial notches	Axial compression	Compression resistance
[121]	CFRP wraps	Pitting corrosion (artificial pits)	Dynamic lateral impact	Stiffness, impact resistance, displacement

**Table 6:** Recommended strengthening and retrofitting materials for different structural applications and service environments [20,26,28,76,97,118,119,120,121].

Application	Best Choice	Considerable	Reasoning
Urban High-Rise	CFRP or AFRP	GFRP	Minimal thickness, excellent durability, no maintenance
Coastal/Marine	AFRP or BFRP	CFRP	Superior chemical resistance, excellent durability
Budget-Limited Bridge	GFRP	Steel Jacket	Lowest cost, acceptable performance, easier installation
Seismic Retrofit	AFRP or FRCM	GFRP	Maximum ductility, energy dissipation capacity
High-Temperature Facility	BFRP or FRCM	CFRP	Thermal stability (400–600 °C), maintain properties
Heritage Building	FRCM or CFRP	AFRP	Minimal dimension increase, esthetic compatibility
Rapid Infrastructure Repair	GFRP	CFRP	Fastest installation, moderate performance
Sustainable/Green Building	BFRP	FRCM	Eco-friendly, low carbon footprint, recyclable

**Table 7:** Comparative evaluation of corrosion mitigation strategies for CFST structures [20,28,41,72,74,75,76,78,97,118,119,120,121].

Mitigation Strategy	Effectiveness (Corrosion Type)	Influence on Structural Behavior	Initial Cost	Maintenance Requirement	Service Life Extension	Practical Implement Ability
Protective Coatings (e.g., epoxy, galvanization)	High for general corrosion; moderate for localized corrosion; limited for circumferential corrosion	Minimal direct structural enhancement; primarily preventive	Low–Moderate	High (periodic reapplication required)	Moderate	Easy to apply; suitable for new and existing structures
Nanocomposite Coatings (e.g., epoxy–silica)	High for general and localized corrosion due to improved impermeability	Negligible structural contribution	Moderate	Low–Moderate	Moderate–High	Moderate complexity; requires controlled application conditions
Cathodic Protection (ICCP/GCP)	Very high for all corrosion types, including pitting and circumferential corrosion	Preserves existing structural capacity; no direct strengthening	High	Moderate (monitoring and system maintenance required)	Very high (up to 30–50 years)	Complex; more suitable for large-scale infrastructure
CFRP Wrapping	High for localized and circumferential corrosion; moderate for general corrosion	Significant improvement in strength, ductility, and buckling resistance	High	Low	High	Easy and fast installation; highly suitable for retrofit
GFRP/AFRP/BFRP Wrapping	Moderate to high depending on fiber type; effective for localized corrosion	Enhanced ductility and moderate strength improvement	Moderate	Low	High	Easy application; cost-effective alternative to CFRP
FRCM Systems	Moderate to high for localized and circumferential corrosion	Improved ductility and compatibility with concrete; moderate strength gain	Moderate	Low	High	Moderate difficulty; good for compatibility and durability
Steel Jacketing	Very high for all corrosion types	Very high strength and stiffness enhancement; improves buckling resistance	Moderate (material)/High (labor)	High (corrosion protection needed)	High	Difficult installation; increases cross-sectional size
Internal Coatings (for steel tube interior)	Moderate for internal/general corrosion; limited for localized external corrosion	No structural enhancement	Low	Low	Moderate	Limited applicability; mainly for new or prefabricated members

Note: The qualitative comparison presented in this table is based on the reported effectiveness, durability, implementation complexity, and practical applicability of the reviewed studies.

## Data Availability

No new data were created or analyzed in this study. Data sharing is not applicable to this article.

## Supplementary Materials

The following supporting information can be downloaded at: https://www.mdpi.com/article/10.3390/ma19153330/s1: PRISMA 2020 checklist.
