# Supplementary material for: Mitigation Strategies for Long-Term Corrosion in CFST Structures: A Systematic Review
Source: Materials (Basel). 2026 Aug 5;19(15):3330. doi: 10.3390/ma19153330 (PMC13467669; doi:10.3390/ma19153330)
Supplement: Supplementary file 1 [file materials-19-03330-s001.zip › materials-4419653-supplementary.pdf]

PRISMA 2020 Checklist

Section and Topic	Item #	Checklist Item	Location where item is reported (Page #)
**TITLE**			
Title	1	Identify the report as a systematic review.	**Page 1** (Title: "...A Systematic Review")
**ABSTRACT**			
Abstract	2	See the PRISMA 2020 for Abstracts checklist.	**Page 1** (Abstract section)
**INTRODUCTION**			
Rationale	3	Describe the rationale for the review in the context of existing knowledge.	**Page 2** (Section 1, Paragraphs 4–11: Explaining corrosion issues and gaps in previous reviews)
Objectives	4	Provide an explicit statement of the objective(s) or question(s) the review addresses.	**Page 2** (Section 1, Paragraph 12: "In response to the increasing durability concerns...")
**METHODS**			
Eligibility criteria	5	Specify the inclusion and exclusion criteria used to select studies.	**Page 2-3** (Section 2, Paragraph 4: English, peer-reviewed, experimental/numerical, carbon steel tubes, etc.)
Information sources	6	Specify all databases, registers, websites,	**Page 2** (Section 2, Paragraph 2: ScienceDirect, ASCE

Section and Topic	Item #	Checklist Item	Location where item is reported (Page #)
		and other sources searched.	Library, SpringerLink, Taylor & Francis, Scopus, Web of Science)
Search strategy	7	Present the full search strategies for all databases, registers, or websites.	**Page 2** (Section 2, Paragraph 1 & Keywords in Fig. 1)
Selection process	8	State the methods used to decide whether a study met the inclusion criteria.	**Page 2** (Section 2, Paragraph 3: "two-phase screening procedure")
Data collection process	9	Specify the methods used to collect data from the reports.	**Page 2** (Section 2, Paragraph 3 & Figure 1)
Data items	10	List and define all outcomes for which data were sought.	**Page 2-3** (Section 2, Paragraph 4 & Section 4: Focus on corrosion behavior and performance degradation)
**RESULTS**			
Study selection	16a	Describe the results of the search and selection process, from the number of records identified to the number of studies included in the review.	**Page 2-3** (Section 2, Paragraphs 1-5 & Figure 1)
	16b	Cite the PRISMA flow diagram.	**Page 2** (Section 2, Paragraph 3: "...as detailed in the PRISMA flow diagram (Fig. 1)")

Section and Topic	Item #	Checklist Item	Location where item is reported (Page #)
Characteristics of included studies	17	Present the characteristics of each included study.	**Page 5** (Table 1: "Comparison Table of Key Corrosion Studies")
Results of individual studies	20	For all outcomes, present for each study summary data for each group.	**Page 5** (Table 1: Key Findings and Performance Reduction)
Discussion	23	Provide a general interpretation of the results in the context of other evidence.	**Page 8** (Section 7: Comparative Evaluation of Mitigation Strategies)
**OTHER INFORMATION**			
Registration and protocol	24a	Provide registration information (or state that it was not registered).	**Page 2** (Section 2, Paragraph 1: "The review protocol was not publicly registered.")
Support / Funding	25	Describe sources of financial or other support for the systematic review.	**Page 1** (Affiliations provided; no specific external funding declared)
